# Securing a Supramolecular Architecture by Tying a Stopper Knot

**DOI:** 10.1002/anie.201803871

**Published:** 2018-05-27

**Authors:** David A. Leigh, Lucian Pirvu, Fredrik Schaufelberger, Daniel J. Tetlow, Liang Zhang

**Affiliations:** ^1^ School of Chemistry University of Manchester Oxford Road Manchester M13 9PL UK

**Keywords:** chemical topology, molecular knots, rotaxanes, supramolecular chemistry

## Abstract

We report on a rotaxane‐like architecture secured by the in situ tying of an overhand knot in the tris(2,6‐pyridyldicarboxamide) region of the axle through complexation with a lanthanide ion (Lu^3+^). The increase in steric bulk caused by the knotting locks a crown ether onto the thread. Removal of the lutetium ion unties the knot, and when the axle binding site for the ring is deactivated, the macrocycle spontaneously dethreads. When the binding interaction is switched on again, the crown ether rethreads over the 10 nm length of the untangled strand. The overhand knot can be retied, relocking the threaded structure, by once again adding lutetium ions.

Macroscopic knots persist and have distinctive properties owing to intra‐strand mechanics and inertia (parts of the strand move only when a force is applied, and in directions determined by the knot structure). In contrast, at the nanoscale, every part of a knotted strand undergoes random thermal motion in all directions. Accordingly, extrapolating knot properties between such disparate length scales, as with machine mechanisms,^1^ will not always be valid.^2^ Although a number of small‐molecule knots have been synthesized,^3^, ^4^ to date the knotting of a strand has only been exploited in functions that have no macroscopic counterpart (anion binding5a,5b and allosterically regulated5c and asymmetric5d catalysis).^1^, ^5^, ^6^ In our everyday world, “stopper knots”^7^ are tied to prevent unreeving (that is, to prevent a strand from passing through a narrow aperture or slipping through another knot^8^). Stopper knots are routinely used to secure ropes for sailing and rock climbing, and threads when sewing. While considering which macroscopic properties of knots might be transferable to the molecular level (and how), we wondered whether the increase in steric bulk that accompanies the tying of a knot could be used to prevent the dethreading of a ring from an axle in a rotaxane‐like architecture.^9^, ^10^, ^11^ In addition to demonstrating that an everyday use of knots can be extrapolated to molecules, the concept is appealing because the locking of the ring on the axle would be accomplished solely through strand entanglement, induced by metal ion coordination, rather than by any functional‐group alterations^12^ (Scheme 1).

**Scheme 1:**
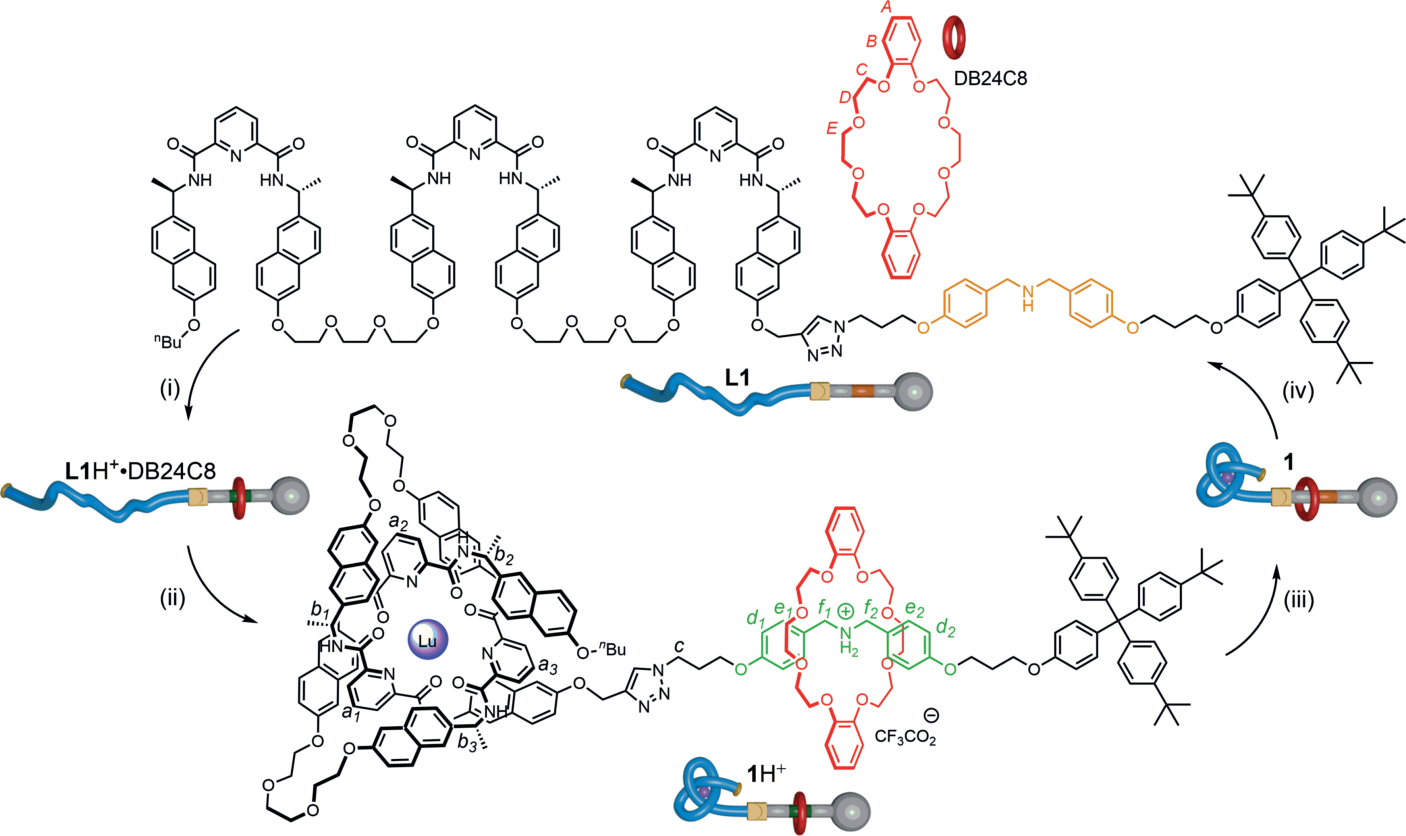
Threading, securing, and releasing a macrocycle on an axle by tying and untying a molecular stopper knot. Reagents and conditions: (i) CF_3_CO_2_H (10 equiv), CD_2_Cl_2_, RT, 48 h, quantitative; (ii) Lu(SO_3_CF_3_)_3_ (1.1 equiv), MeCN‐*d*_3_, 80 °C, 16 h, 90 % ; (iii) Et_3_N (10 equiv), CD_2_Cl_2_, RT, 1 h, quantitative; (iv) Et_4_NF (10 equiv), CD_2_Cl_2_, RT, 5 min, quantitative.

We designed a rotaxane system to explore this idea by using an axle bearing an ammonium group, an excellent binding site for dibenzo‐24‐crown‐8 (DB24C8),^13^ terminated at one end by a bulky trityl derivative and at the other by a tris(2,6‐pyridinedicarboxamide)^14^ region (**1**H^+^, Scheme 1). Tris(2,6‐pyridinedicarboxamide) ligands can be tied5d into overhand knots^15^ through coordination to lanthanide(III) ions.^14^, ^16^, ^17^ We found it convenient to assemble the complete axle as a rotaxane (Scheme 2; see also the Supporting Information). Ligand building block **L2** was treated with Lu(CF_3_SO_3_)_3_ in MeCN at 80 °C to generate overhand knot Λ‐**L2**⋅Lu (Λ refers to the handedness of the knot;^18^ for steric reasons, only the Λ knot can form from the *R*,*R*,*R*,*R*,*R*,*R* enantiomer of **L1**).5d, 19 After 16 h, the knotted lanthanide complex was the only species evident by electrospray ionization mass spectrometry (ESI‐MS) and ^1^H NMR spectroscopy (Scheme 2, step (i)). Treatment of azide **L3**H^+^ with DB24C8 in the presence of trifluoroacetic acid generated pseudorotaxane **L3**H^+^⋅DB24C8 (Scheme 2, step (ii)). A CuAAC reaction of threaded complex **L3**H^+^⋅DB24C8 with Λ‐**L2**⋅Lu generated rotaxane architecture **1**H^+^ in 41 % yield, following purification by size exclusion chromatography (Scheme 2, step (iii)).

**Scheme 2:**
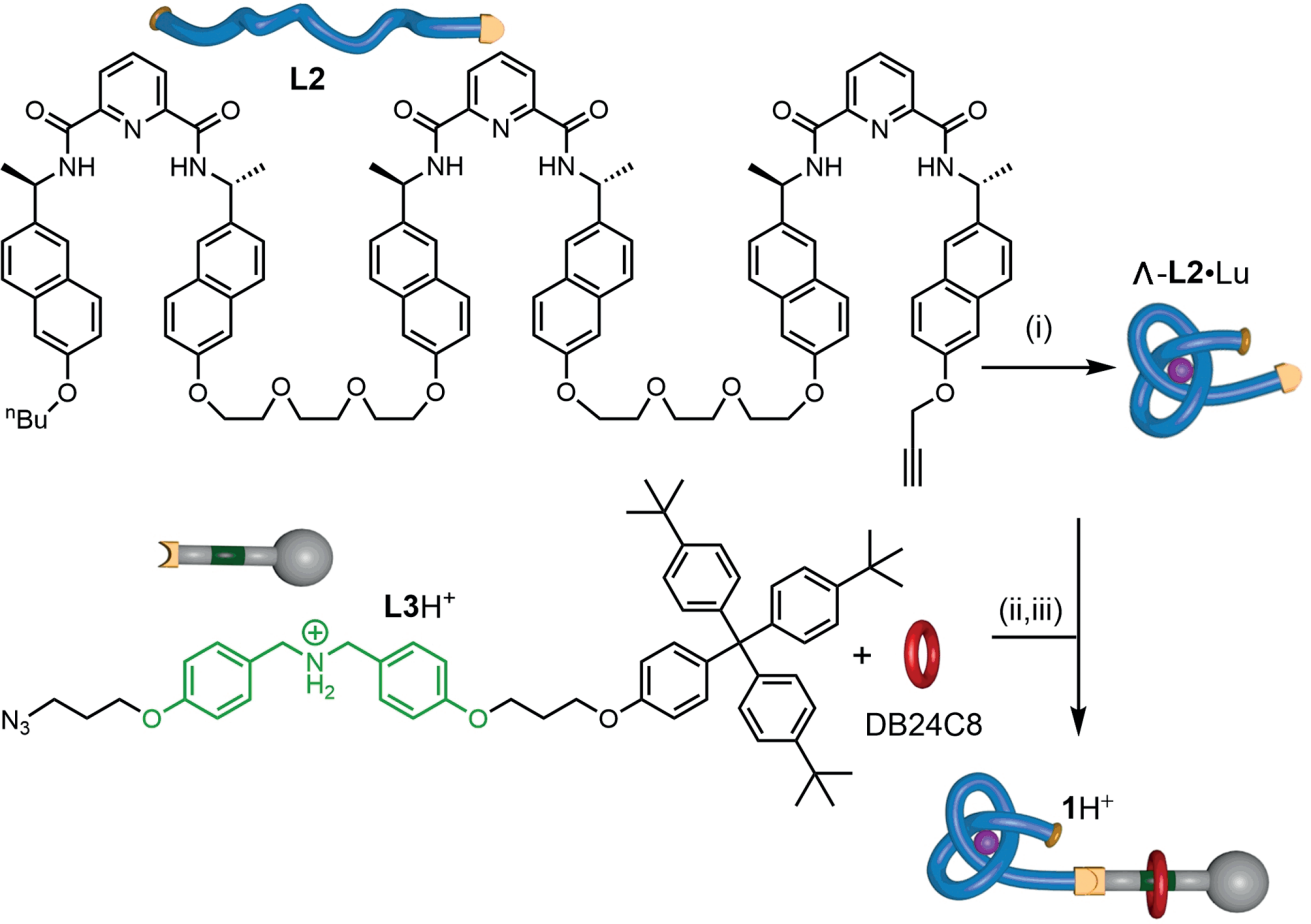
Synthesis of rotaxane architecture **1**H^+^. Reagents and conditions: (i) Lu(CF_3_SO_3_)_3_ (1 equiv), MeCN, 80 °C, 16 h, 75 %; (ii) **L3**H^+^, DB24C8 (4 equiv), CF_3_COOH (10 equiv), CH_2_Cl_2_, RT, 10 min; then (iii) Λ‐**L2**⋅Lu, (CH_3_CN)_4_Cu(CF_3_SO_3_) (2 equiv), MeCN/MeOH (1:1), RT, 20 h, 41 %.

^1^H NMR spectroscopy (600 MHz, 298 K, MeCN‐*d*_3_) confirmed that both the knotted conformation of the strand and the threaded structure are maintained in **1**H^+^ (Figure 1). The overhand knot is evident from the upfield shift of the pyridine H_a1_/H_a2_ protons (see the Supporting Information, Spectrum S11), which is due to π‐stacking in the knotted conformation,5d and the diastereotopic splitting of various axle signals caused by the asymmetry of the knot (Spectrum S18). The benzylic protons H_f1_ and H_f2_ appear at *δ*=4.55 ppm in **1**H^+^ (Figure 1 b), which corresponds to a downfield shift of 0.40 ppm compared to the macrocycle‐free knotted axle Λ‐**L1**H^+^⋅Lu (Figure 1 a). ESI‐MS analysis of **1**H^+^ was consistent with the rotaxane structure (*m*/*z* 830.1 [**1**H^+^]^4+^, 1156.0 [**1**H^+^][CF_3_SO_3_]^3+^, 1808.6 [**1**H^+^][CF_3_SO_3_]_2_^2+^; see Figure S1).

**Figure 1:**
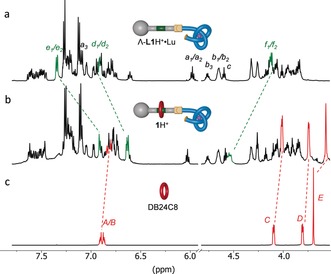
Partial ^1^H NMR spectra (600 MHz, MeCN‐*d*_3_, 298 K) showing the threaded structure of **1**H^+^. a) Λ‐**L1**H^+^⋅Lu. b) **1**H^+^. c) DB24C8.

To confirm that the overhand knot blocks dethreading of the macrocycle, the dibenzylammonium binding site of **1**H^+^ was “switched off” by deprotonation with triethylamine, forming **1** (Scheme 1, step (iii); Et_3_N, CD_2_Cl_2_, RT, 1 h; see also Figures S2 and S3).^20^ In CD_2_Cl_2_, the ^1^H NMR spectrum of **1** is broad for internal regions of the axle (Figure 2 a), suggesting slow dynamics of ring movement between various sites and conformations.^21^ In MeCN‐*d*_3_, the macrocycle samples much of the axle as evidenced by modest shifts in the ^1^H NMR resonances of protons all along the length of the thread (Figure S3). X‐ray crystal structures of knotted tris(2,6‐pyridinedicarboxamide) lanthanide complexes5d, 16 indicate that the tied knot has a diameter of approximately 2 nm, whereas the aperture of DB24C8 is <1 nm. Accordingly, with the binding site for the crown ether deactivated, the rotaxane architecture of **1** is still kinetically stable; at room temperature, **1** showed no signs of dethreading over several weeks in CD_2_Cl_2_ or MeCN‐*d_3_* solution.

**Figure 2:**
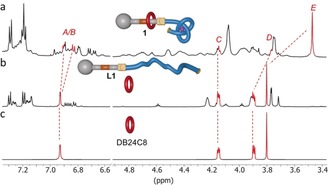
Triggered dethreading of **1**. a) Partial ^1^H NMR spectrum (600 MHz, CD_2_Cl_2_, 298 K) of **1**. b) Partial ^1^H NMR spectrum (600 MHz, CD_2_Cl_2_, 298 K) recorded 5 min after addition of Et_4_NF (10 equiv) to **1**. c) Partial ^1^H NMR spectrum (600 MHz, CD_2_Cl_2_, 298 K) of DB24C8.

The overhand knot of **1** was untied, and the macrocycle released, by treatment of the deprotonated rotaxane with tetraethylammonium fluoride (10 equiv) in CD_2_Cl_2_ (Scheme 1, step (iv)). ^1^H NMR analysis indicated quantitative dethreading of the crown ether within 5 min of the addition of the fluoride salt (Figure 2 b). MALDI mass spectrometry confirmed the presence of **L1** (*m*/*z* 2716.4, [*M*+Na^+^]; Figure S6), with no evidence of a demetalated pseudorotaxane. This indicates that with the binding site deactivated, ring dethreading occurs as soon as the knot is untied. The quantitative dethreading further confirms the complete deprotonation of **1** (the DB24C8–secondary ammonium equilibrium in CD_2_Cl_2_ strongly favours the pseudorotaxane).20b

The presence of the knot also prevents threading onto the axle: Treatment of the protonated knotted thread Λ‐**L1**H^+^⋅Lu with DB24C8 failed to generate any [2]rotaxane (Scheme S9).

Finally, we demonstrated that the ring could thread over the about 10 nm length of the untangled strand^22^ and the rotaxane architecture subsequently be secured by tying the stopper knot (Scheme 1, steps (i) and (ii)). Addition of trifluoroacetic acid to a solution of DB24C8 (10 equiv) and **L1** in CD_2_Cl_2_ afforded the pseudorotaxane complex **L1**H^+^ over 48 h (Figures 3 a and S7). Excess trifluoroacetic acid was removed under reduced pressure, Lu(CF_3_SO_3_)_3_ (1.1 equiv in MeCN‐*d*_3_) was added, and the solution heated at 80 °C. After 16 h, both ^1^H NMR and ESI‐MS analysis confirmed the formation of **1**H^+^ in approximately 90 % yield (determined by ^1^H NMR analysis; Figures 3 b and S8).

**Figure 3:**
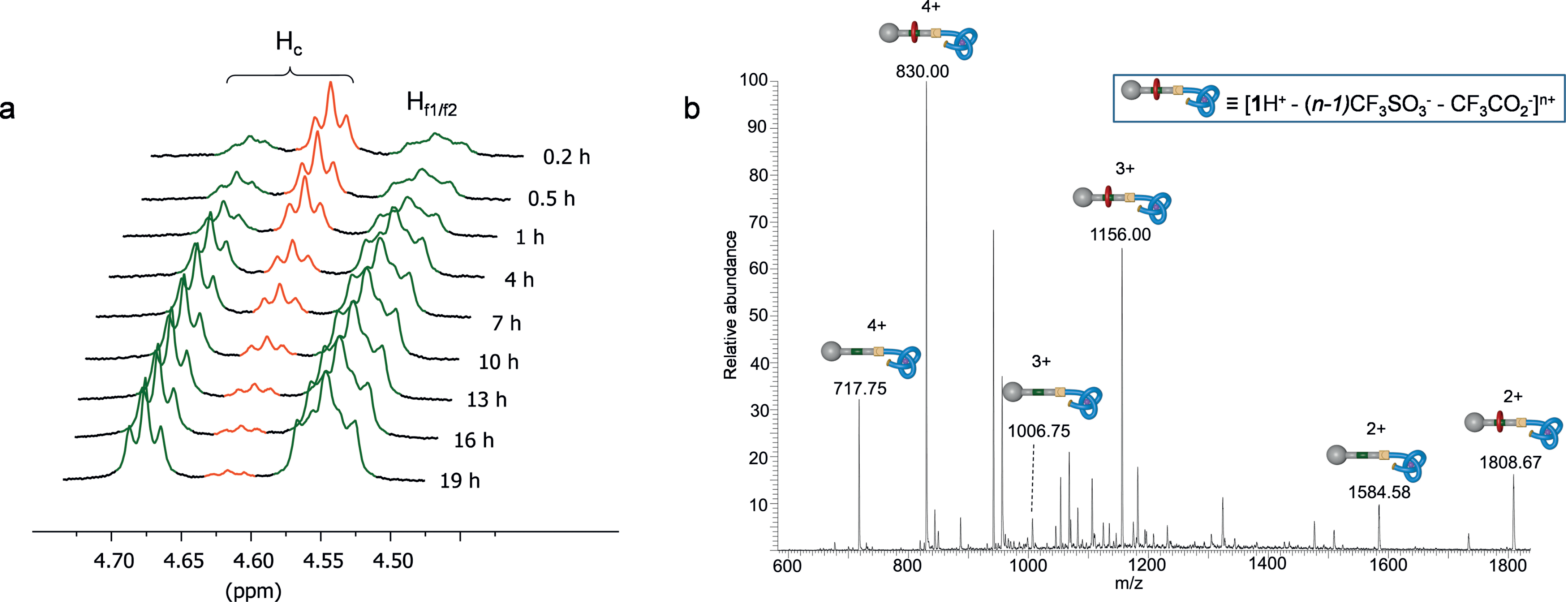
Securing a rotaxane architecture (**1**H^+^) by threading a ring (DB24C8) onto an axle (**L1**) and tying a molecular stopper knot. a) Partial ^1^H NMR spectrum (600 MHz, MeCN‐*d*_3_, 298 K) stack plot of the threading process to form **L1**H^+^⋅DB24C8 (Scheme 1, step (i)), showing signals for H_c_ and H_f1_/H_f2_. b) Detection of knotted, threaded **1**H^+^ by ESI‐MS (positive mode) following in situ tying of an overhand stopper knot in **L1**H^+^⋅DB24C8 through coordination to Lu^3+^ (Scheme 1, step (ii)).

In conclusion, we have demonstrated that despite the fundamental differences in mechanical behaviour across length scales, a synthetic molecular knot can perform a mechanical function analogous to a mechanical function performed by macroscopic knotting. Once a thermodynamic driving force has been used to thread the molecular ring onto the axle, addition of a lanthanide ion ties the molecular stopper knot, locking the ring on the axle even after the ring binding site has been deactivated. The knot can subsequently be untied, and the ring released from the axle, by removing the metal ion. Release of the macrocycle requires no change to the covalent structure of the molecule. The difference in the timescales required for tying (16 hours at 80 °C) and untying (<5 min at room temperature) the stopper knot is particularly striking. Such processes may prove useful in the development of functional knotted molecules and materials.

## Conflict of interest

The authors declare no conflict of interest.

## Supporting information

As a service to our authors and readers, this journal provides supporting information supplied by the authors. Such materials are peer reviewed and may be re‐organized for online delivery, but are not copy‐edited or typeset. Technical support issues arising from supporting information (other than missing files) should be addressed to the authors.

Supplementary
