# Supplementary material for: Securing a Supramolecular Architecture by Tying a Stopper Knot
Source: Angew Chem Int Ed Engl. 2018 May 27;57(33):10484–8. doi: 10.1002/anie.201803871 (PMC6099318; doi:10.1002/anie.201803871)
Supplement: Supplementary file 1 — Supplementary [file ANIE-57-10484-s001.pdf]

Angewandte Chemie
Eine Zeitschrift der Gesellschaft Deutscher Chemiker

Supporting Information

Securing a Supramolecular Architecture by Tying a Stopper Knot

David A. Leigh, Lucian Pirvu, Fredrik Schaufelberger, Daniel J. Tetlow, and Liang Zhang*

anie_201803871_sm_miscellaneous_information.pdf

Table of Contents

S1. Abbreviations……………………………………………………………………..……..…..S3

S2. General Experimental ………………………………………….................................S4

S3. Experimental Procedures ……………………………………………………….……..S5

S3.1 Synthesis of **L2**……..……..………………………………………………………….S5

S3.2 Synthesis of Λ-**L2**•Lu …………………………………………………..……………..S10

S3.3 Synthesis of **L3**H$^+$………..S12

S3.4 Synthesis of **1**H$^+$…………………………………………………………………..……..S14

S3.5 Synthesis of **1**…………………………………………………………….………..…...S17

S3.6. Synthesis of Λ-**L1**H$^+$•Lu ……………………………...S20

S3.7. Dethreading of **1**……………………………………………………………..…………S23

S3.8. Threading and remetallation of **L1**……………………….......................................S26

S3.9. Attempted rethreading of metallated Λ-**L1**H$^+$•Lu ……………….............................S29

S4. NMR Spectra ……………………………………………..S30

S5. Supplementary Information References…………………………………………….....S43

S1. Abbreviations

Abbreviations: Boc *tert*-butoxycarbonyl carbamate; DB24C8 dibenzo-24-crown-8; DMF *N*,*N*-dimethylformamide; DMSO dimethylsulfoxide; EDTA ethylenediaminetetraacetic acid; Et_3N triethylamine; MeCN acetonitrile; MsCl Methanesulfonyl chloride; quant. quantitative; r.t. room temperature; TEAF tetraethylammonium fluoride; TFA trifluoroacetic acid; THF tetrahydrofuran; TLC thin layer chromatography.

S2. General Experimental

Unless stated otherwise, reagents were obtained from commercial sources and used without purification. Reactions were carried out in anhydrous solvents and under an N_2 atmosphere. Anhydrous solvents were obtained by passing the solvent through an activated alumina column on a Phoenix SDS (solvent drying system; JC Meyer Solvent Systems, CA, USA). **S1**[1] and **S7**[2] were synthesized according to previously reported procedures. ^{1}H NMR spectra were recorded on a Bruker Avance III instrument with an Oxford AS600 magnet equipped with a cryoprobe [5mm CPDCH ^{13}C-^{1}H/D] (600 MHz). Chemical shifts are reported in parts per million (ppm) from high to low frequency using the residual solvent peak as the internal reference ($CDCl_3$ = 7.26 ppm, CD_2Cl_2 = 5.32 ppm, CD_3OD = 3.31 ppm, $(CD_3)_2SO$ = 2.50 ppm and CD_3CN = 1.94 ppm). All ^{1}H resonances are reported to the nearest 0.01 ppm. The multiplicity of ^{1}H signals are indicated as: s = singlet; d = doublet; t = triplet; q = quartet; m = multiplet; br = broad; app = apparent; or combinations of thereof. Coupling constants (*J*) are quoted in Hz and reported to the nearest 0.1 Hz. Where appropriate, averages of the signals from peaks displaying multiplicity were used to calculate the value of the coupling constant. ^{13}C NMR spectra were recorded on the same spectrometer with the central resonance of the solvent peak as the internal reference (CD_3CN = 118.26 ppm, $CDCl_3$ = 77.16 ppm, CD_2Cl_2 = 54.00 ppm, CD_3OD = 49.00 ppm and $(CD_3)_2SO$ = 39.52 ppm). All ^{13}C resonances are reported to the nearest 0.1 ppm, except in cases to aid the differentiation of closely resolved signals (which are reported to the nearest 0.01 ppm). DEPT, COSY, HSQC and HMBC experiments were used to aid structural determination and spectral assignment. Fully characterized compounds were chromatographically homogeneous. Flash column chromatography was carried out using Silica 60 Å (particle size 40–63 μm, Sigma Aldrich, UK) as the stationary phase. Size exclusion chromatography was carried out using Bio-Beads™ S-X1 Support beads as the stationary phase. TLC was performed on precoated silica gel plates (0.25 mm thick, 60 F_{254}, Merck, Germany) and visualized using both short and long wave ultraviolet light in combination with standard laboratory stains (basic potassium permanganate, acidic ammonium molybdate and ninhydrin). Low resolution ESI mass spectrometry was performed with a Thermo Scientific LCQ Fleet Ion Trap Mass Spectrometer or an Agilent Technologies 1200 LC system with either an Agilent 6130 single quadrupole MS detector or an Advion Expression LCMS single quadrupole MS detector. High-resolution mass spectrometry was carried out by the EPSRC National Mass Spectrometry Service Centre (Swansea, UK) or by staff at the Mass Spectrometry Service, School of Chemistry, The University of Manchester. Melting points (MP) were determined using a Büchi M-565 apparatus and are uncorrected.

S3. Experimental Procedures

3.1 Synthesis of **L2**

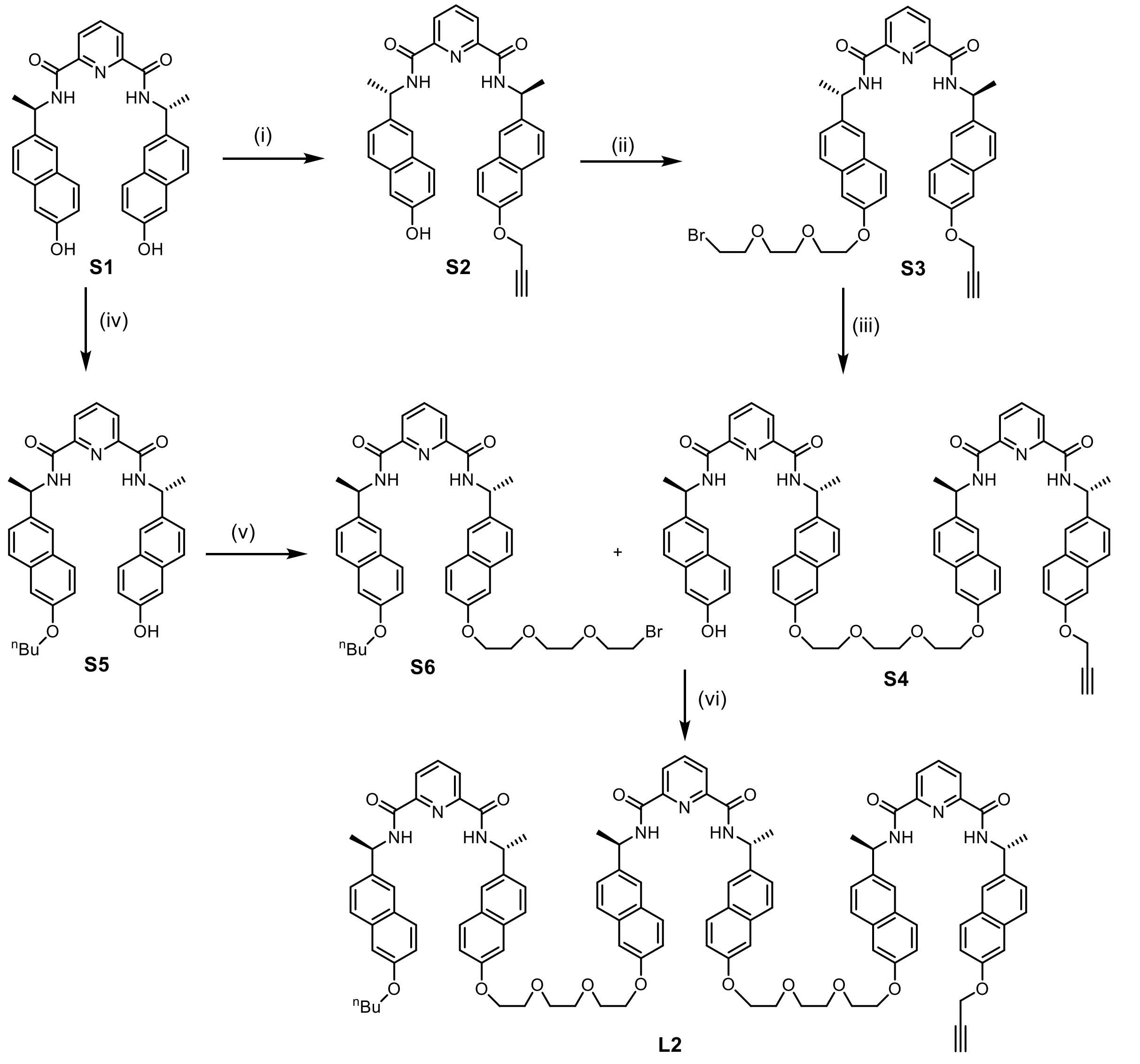

Scheme S1: Synthesis of **L2**. Reagents and conditions: (i) propargyl bromide, K_2CO_3, DMF, 80 °C, 2 h, 41%. (ii) 1,2-bis(2-bromoethoxy)ethane, K_2CO_3, DMF, 80 °C, 1 h, 80%. (iii) **S1**, K_2CO_3, DMF, 80 °C, 16 h, 32%. (iv) 1-bromobutane, K_2CO_3, DMF, 80 °C, 2 h, 39%. (v) 1,2-bis(2-bromoethoxy)ethane, K_2CO_3, DMF, 80 °C, 1 h, 76%. (vi) K_2CO_3, DMF, 80 °C, 16 h, 88%.

S2

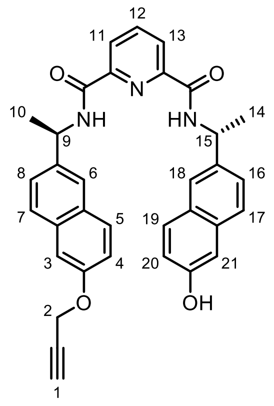

To a solution of **S1**[1] (1.03 g, 2.03 mmol) and potassium carbonate (0.28 g, 2.03 mmol) in degassed DMF[a] (20 mL) was added propargyl bromide (80 % in toluene, 0.22 mL, 2.03 mmol). The reaction was stirred for 2 hours at 80 °C. The mixture was allowed to cool to room temperature and concentrated under reduced pressure. Purification by flash column chromatography (SiO_2, CH_2Cl_2:EtOAc 2:1) yielded **S2** (464 mg, 41%) as a colorless solid. **MP** 146–148 °C; **^{1}H NMR** (600 MHz, CD_3OD) δ 8.28 (d, *J* = 7.9 Hz, 2H, $H_{11,13}$), 8.13 (t, *J* = 7.7 Hz, 1H, H_{12}), 7.80 (s, 1H, $H_{6/18}$), 7.77 (s, 1H, $H_{6/18}$), 7.74 (d, *J* = 8.7 Hz, 2H, $H_{5,7}$), 7.68 (d, *J* = 8.8 Hz, 1H, H_{19}), 7.62 (d, *J* = 8.6 Hz, 1H, H_{17}), 7.52 (dd, *J* = 8.5, 1.5 Hz, 1H, H_8), 7.46 (dd, *J* = 8.5, 1.5 Hz, 1H, H_{16}), 7.29 (d, *J* = 2.3 Hz, 1H, H_3), 7.14 (dd, *J* = 8.9, 2.5 Hz, 1H, H_4), 7.08 (d, *J* = 1.8 Hz, 1H, H_{21}), 7.05 (dd, *J* = 8.8, 2.3 Hz, 1H, H_{20}), 5.50 – 5.43 (m, 2H, $H_{9,15}$), 4.83 (d, *J* = 2.3 Hz, 2H, H_2), 2.96 (t, *J* = 2.3 Hz, 1H, H_1), 1.70 (d, *J* = 6.8 Hz, 3H, $H_{14/10}$), 1.69 (d, *J* = 6.8 Hz, 3H, $H_{14/10}$); **^{13}C NMR** (151 MHz, CD_3OD) δ 163.97, 163.93, 155.65, 155.11, 149.31, 149.27, 138.93, 138.82, 137.63, 134.22, 133.64, 129.15, 129.07, 129.04, 128.26, 127.00, 126.27, 125.08, 124.83, 124.82, 124.02, 123.98, 118.56, 118.14, 108.38, 106.99, 78.35, 75.43, 55.25, 48.83, 20.27, 20.24. **HRMS** (ESI^+): Calcd. for $C_{34}H_{29}N_3O_4K^+$: 582.1790, found 582.1796 $[M+K]^+$. *Proton signals of OH and NH are not observed. *2 carbon signals not resolved due to overlap of signals in spectrum.

S3

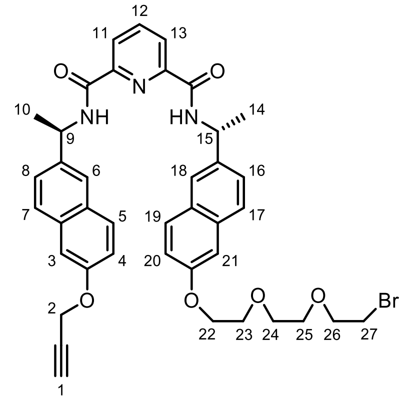

To a solution of **S2** (0.46 g, 0.85 mmol) and potassium carbonate (0.6 g, 4.4 mmol) in degassed DMF (20 mL) was added 1,2-bis(2-bromoethoxy)ethane (0.44 mL, 2.67 mmol). The reaction was stirred for 1 hour at 80 °C. The mixture was allowed to cool to room temperature and concentrated under reduced pressure. Purification by flash column chromatography (SiO_2, CH_2Cl_2:EtOAc 2:1) yielded **S3** (508 mg, 80%) as a colourless solid. **MP** 95–97 °C; **^{1}H NMR** (600 MHz, $CDCl_3$) δ 8.36 (dd, *J* = 7.7, 1.4 Hz, 2H, $H_{11,13}$), 8.03 (t, *J* = 7.8 Hz, 1H, H_{12}), 7.86 (d, *J* = 7.9 Hz, 2H, NH), 7.73 (app d, *J* = 7.1 Hz, 2H, $H_{6,18}$), 7.69 (d, *J* = 8.8 Hz, 1H, $H_{5/7/17/19}$), 7.67 (d, *J* = 9.0 Hz, 1H, $H_{5/7/17/19}$), 7.65 (d, *J* = 8.6 Hz, 1H, $H_{5/7/17/19}$), 7.63 (d, *J* = 8.6 Hz, 1H, $H_{5/7/17/19}$), 7.43 (td, *J* = 8.5, 1.7 hz, 2H, $H_{8,16}$), 7.21 (d, *J* = 2.3 Hz, 1H, H_{21}), 7.20 (dd, *J* = 9.0,

[a] Degassed by sparging with nitrogen for 30 minutes.

2.4 Hz, 2H, $H_{4,20}$) 7.11 (d, J = 2.3 Hz, 1H, H_3), 5.43 (quintet, J = 7.3 Hz, 2H, $H_{9,15}$), 4.82 (d, J = 2.3 Hz, 2H, H_2), 4.27 (app t, J = 4.6 Hz, 2H, H_{22}), 3.95 (app t, J = 4.6 Hz, 2H, H_{23}), 3.82 (t, J = 6.2 Hz, 2H, H_{26}), 3.77 (app dd, J = 6.6, 2.6 Hz, 2H, $H_{24/25}$), 3.71 (app dd, J = 6.2, 2.7 Hz, 2H, $H_{24/25}$), 3.69 (t, J = 6.3 Hz, 2H, H_{27}), 2.56 (t, J = 2.3 Hz, 1H, H_1), 1.65 (d, J = 6.8 Hz, 6H, $H_{10,14}$); **^{13}C NMR** (151 MHz, $CDCl_3$) δ 162.58, 162.57, 157.02, 155.69, 148.83, 148.80, 139.12, 138.28, 137.86, 133.90, 133.68, 129.58, 129.38, 129.15, 128.86, 127.76, 127.59, 125.19, 125.01, 124.91, 124.63, 124.61, 119.60, 119.31, 107.35, 106.61, 78.38, 75.85, 71.27, 70.89, 70.63, 69.84, 67.48, 55.88, 49.09, 30.97, 30.36, 21.65, 21.62. **HRMS** (ESI$^+$): Calcd. for $C_{40}H_{40}N_3O_6BrK^+$: 776.1732, found 776.1734 $[M+K]^+$. *1 carbon signal not resolved due to overlap of signals in spectrum.

S4

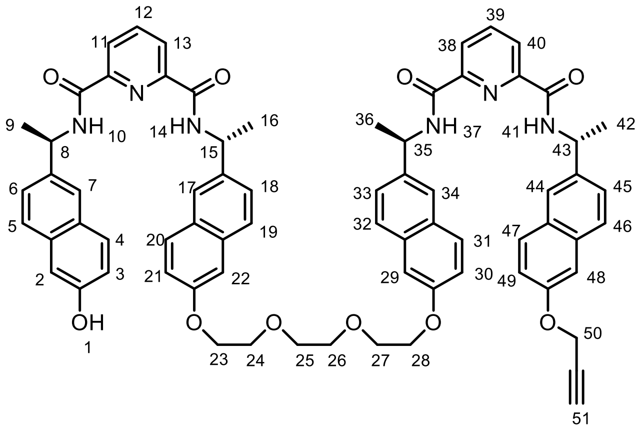

To a solution of **S3** (0.48 g, 0.64 mmol) and potassium carbonate (0.089 g, 0.64 mmol) in degassed DMF (40 mL) was added **S1** (0.32 g, 0.64 mmol). The reaction was stirred overnight at 80 °C. The reaction mixture was allowed to cool to room temperature and concentrated under reduced pressure. Purification by flash column chromatography (SiO_2, EtOAc) yielded **S4** (243 mg, 32 %) as an off-white solid. **MP** 152–156 °C; **^{1}H NMR** (600 MHz, DMSO-d_6) δ 9.74 (s, J = 7.7, 1.4 Hz, 1H, H_1), 9.53 – 9.46 (m, 4H, $H_{10,14,37,41}$), 8.29 – 8.24 (m, 4H, $H_{11,13,38,40}$), 8.23 – 8.18 (m, 2H, $H_{12,39}$), 7.91 – 7.78 (m, 10H, $H_{7,17,19,20,31,32,34,44,46,47}$), 7.76 (d, J = 8.8 Hz, 1H, H_4), 7.7 (d, J = 8.6 Hz, 1H, H_5), 7.62 (dd, J = 8.6, 1.5 Hz, 1H, H_{45}), 7.60 (app dt, J = 8.5, 1.7 Hz, 2H, $H_{18,33}$), 7.53 (dd, J = 8.5, 1.5 Hz, 1H, H_6), 7.4 (d, J = 2.4 Hz, 1H, H_{48}), 7.33 (d, J = 2.0 Hz, 2H, $H_{22,29}$), 7.22 (dd, J = 8.9, 2.5 Hz, 1H, H_{49}), 7.18 (dd, J = 8.9, 2.3 Hz, 2H, $H_{21,30}$), 7.13 (s, 1H, H_2), 7.1 (dd, J = 8.8, 2.4 Hz, 1H, H_3), 5.44 (m, 4H, $H_{8,15,35,43}$), 4.94 (d, J = 2.3 Hz, 2H, H_{50}), 4.23 (app t, J = 9.0 Hz, 4H, $H_{23,28}$), 3.85 (app t, J = 8.9 Hz, 4H, $H_{24,27}$), 3.69 (s, 4H, $H_{25,26}$), 3.63 (t, J = 2.3 Hz, 1H, H_{51}), 1.74 – 1.68 (m, 12H, $H_{9,16,36,42}$); **^{13}C NMR** (151 MHz, DMSO-d_6) δ 207.03, 163.33, 156.73, 155.63, 155.44, 149.60, 139.89, 139.81, 139.43, 138.43, 134.09, 133.75, 133.48, 129.81, 129.70, 128.95, 128.67, 127.87, 127.48, 127.38, 126.70, 126.16, 126.02, 125.79, 125.24, 124.53, 119.35, 119.26, 119.16, 108.98, 107.87, 106.99, 79.66, 78.82, 70.44, 69.41, 67.61, 55.94, 48.59, 31.16, 22.15. **HRMS** (ESI$^+$): Calcd. for $C_{71}H_{66}N_6O_{10}K^+$: 1201.4472, found

1201.4426 $[M+K]^+$. *Due to high degree of apparent symmetry within **S4** many ^{13}C signals overlap.

S5

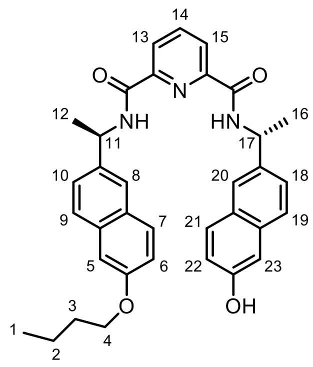

To a solution of **S1** (0.34 g, 0.67 mmol) and potassium carbonate (0.092 g, 0.67 mmol) in degassed DMF (50 mL) was added 1-bromobutane (72 µL, 0.67 mmol). The reaction was stirred for 2 hours at 80 °C. The mixture was allowed to cool to room temperature and concentrated under reduced pressure. Flash column chromatography (SiO_2, CH_2Cl_2:EtOAc 2:1) gave **S5** (148 mg, 39%) as a colorless solid. **MP** 152–155 °C; **^{1}H NMR** (600 MHz, CD_3OD) δ 8.27 (d, *J* = 7.8 Hz, 2H, $H_{13,15}$), 8.11 (t, *J* = 7.8 Hz, 1H, H_{14}), 7.76 (s, 1H, H_8), 7.75 (s, 1H, H_{20}), 7.71 – 7.66 (m, 3H, $H_{7,9/19,21}$), 7.61 (d, *J* = 8.6 Hz, 1H, $H_{9/19}$), 7.48 (dd, *J* = 8.5, 1.6 Hz, 1H, $H_{10/18}$), 7.45 (dd, *J* = 8.6, 1.7 Hz, 1H, $H_{10/18}$), 7.16 (d, *J* = 2.3 Hz, 1H, H_5), 7.10 (dd, *J* = 8.9, 2.5 Hz, 1H, H_6), 7.08 (d, *J* = 2.3 Hz, 1H, H_{23}), 7.05 (dd, *J* = 8.8, 2.5 Hz, 1H, H_{22}), 5.44 (quintet, *J* = 7.2 Hz, 2H, $H_{11,17}$), 4.07 (t, *J* = 6.6 Hz, 2H, H_4), 1.83 – 1.78 (m, 2H, H_3), 1.68 (d, *J* = 7.0 Hz, 3H, $H_{12/16}$), 1.67 (d, *J* = 7.0 Hz, 3H, $H_{12/16}$), 1.54 (sextet, *J* = 7.4 Hz, 2H, H_2), 1.01 (t, *J* = 7.8 Hz, 3H, H_1); **^{13}C NMR** (151 MHz, CD_3OD) δ 163.94, 163.92, 157.18, 155.11, 149.28, 149.26, 138.90, 138.28, 137.62, 134.21, 133.96, 129.04, 128.88, 128.77, 128.25, 126.83, 126.26, 124.90, 124.84, 124.80, 124.01, 123.95, 118.80, 118.14, 108.38, 105.95, 67.30, 48.83, 48.81, 31.13, 20.27, 20.24, 18.99, 12.82; **HRMS** (ESI^+): Calcd. for $C_{35}H_{35}N_3O_4K^+$: 600.2259, found 600.2266 $[M+K]^+$. *Proton signals of OH and NH are not observed. *1 carbon signal not resolved due to overlap of signals in spectrum

S6

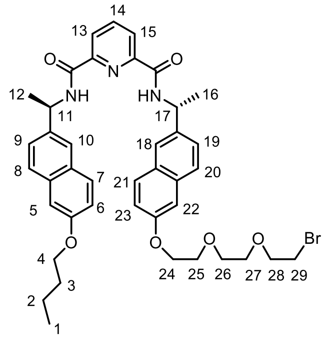

To a solution of **S5** (0.14 g, 0.25 mmol) and potassium carbonate (0.18 g, 1.32 mmol) in degassed DMF (7 mL) was added 1,2-bis(2-bromoethoxy)ethane (0.13 ml, 0.8 mmol). The reaction was stirred for 1 h at 80 °C. The mixture was allowed to cool to room temperature and concentrated under reduced pressure. Purification by flash column chromatography (SiO_2, CH_2Cl_2: EtOAc 2:1) yielded **S6** (0.143 g, 76%) as a colorless solid. **MP** 95–98 °C; **^{1}H NMR** (600 MHz, $CDCl_3$) δ 8.36 (d, *J* = 7.6 Hz, 1H, $H_{13/15}$), 8.35 (d, *J* = 7.9 Hz, 1H, $H_{13/15}$). 8.03 (t, *J* = 7.8 Hz, 1H, H_{14}), 7.86 (dd, *J* = 7.9, 1.0 Hz, 2H, NH), 7.73 (s, 2H,

$H_{10,18}$), 7.67 (dd, *J* = 9.0, 1.6 Hz, 2H, $H_{7,21}$), 7.65 (d, *J* = 8.4 Hz, 1H, $H_{8/9/19/20}$), 7.63 (d, *J* = 8.4 Hz, 1H, $H_{8/9/19/20}$), 7.42 (d, *J* = 8.5 Hz, 2H, $H_{8/9/19/20}$), 7.19 (dd, *J* = 8.9, 2.5 Hz, 1H, $H_{6/22}$), 7.16 (dd, *J* = 8.9, 2.4 Hz, 2H, $H_{6/22}$), 7.11 (app t, *J* = 2.4 Hz, 2H, $H_{5,23}$), 5.47 – 5.40 (m, 2H, $H_{11,17}$), 4.27 – 4.25 (m, 2H, H_{24}), 4.08 (t, *J* = 6.6 Hz, 2H, H_4), 3.96 – 3.94 (m, 2H, H_{25}), 3.82 (t, *J* = 6.3 Hz, 2H, H_{28}), 3.77 (dd, *J* = 6.4, 3.9 Hz, 2H, H_{26}), 3.72 (dd, *J* = 5.2, 3.8 Hz, 2H, H_{27}), 3.47 (t, *J* = 6.3 Hz, 2H, H_{29}), 1.87 – 1.80 (quintet, *J* = 6.6 Hz, 2H, H_3), 1.66 (d, *J* = 6.8 Hz, 2H, $H_{12/16}$), 1.65 (d, *J* = 6.8 Hz, 2H, $H_{12/16}$), 1.57 – 1.51 (sextet, *J* = 7.38, 2H, H_2), 1.01 (t, *J* = 7.4 Hz, 3H, H_1); **^{13}C NMR** (151 MHz, $CDCl_3$) δ 162.56, 157.40, 157.00, 148.84, 148.80, 139.11, 137.91, 137.65, 134.04, 133.89, 129.38, 129.30, 128.87, 128.69, 127.59, 127.51, 125.18, 124.89, 124.86, 124.60, 119.61, 119.57, 106.61, 106.42, 71.27, 70.89, 70.63, 70.55, 69.84, 67.79, 67.48, 49.07, 31.31, 30.35, 21.67, 21.61, 19.34, 13.93. **HRMS** (ESI^+): Calcd. for $C_{41}H_{46}N_3O_6BrK^+$: 794.2202, found 794.2206 $[M+K]^+$. *3 carbon signals not resolved due to overlap of signals in spectrum

L2

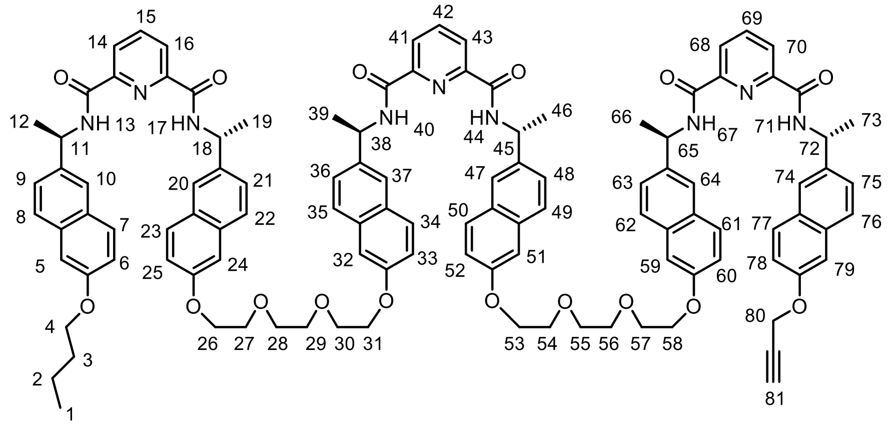

To a solution of **S4** (0.22 g, 0.19 mmol) and potassium carbonate (0.15 g, 1.08 mmol) in degassed DMF (15 mL) was added a solution of **S6** (0.14 g, 0.19 mmol) in DMF (20 mL). The reaction was stirred overnight at 80 °C. The mixture was allowed to cool to room temperature and concentrated under reduced pressure. Purification by flash column chromatography (SiO_2, EtOAc 100 %, then CH_2Cl_2:MeOH 9:1) yielded **L2** (0.311 g, 88%) as a colorless solid. **MP** 126–129 °C; **^{1}H NMR** (600 MHz, DMSO-*d$_6$*) δ 9.52 – 9.45 (m, 6H, $H_{13,17,40,44,67,71}$), 8.27 – 8.24 (m, 6H, $H_{14,16,41,43,68,70}$), 8.23 – 8.18 (m, 3H, $H_{15,42,69}$), 7.91 – 7.78 (m, 18H, $H_{7,8,10,20,22,23,34,35,37,47,49,50,61,62,64,74,76,77}$), 7.64 – 7.57 (m, 6H, $H_{9,21,36,48,63,75}$), 7.40 (d, 2.3 Hz, 1H, $H_{5/79}$), 7.34 – 7.30 (s, 5H, $H_{5/79,24,32,51,59}$), 7.21 (dd, *J* = 8.9, 2.5 Hz, 1H, $H_{6/78}$), 7.19 – 7.15 (m, 5H, $H_{6/78,25,33,52,60}$), 5.47 – 5.39 (m, 6H, $H_{11,18,38,45,65,72}$), 4.94 (d, *J* = 2.3 Hz, 2H, H_{80}), 4.22 (app t, *J* = 4.4 Hz, 8H, $H_{26,31,53,58}$), 4.10 (t, *J* = 6.5 Hz, 2H, H_4), 3.84 (app t, *J* = 4.5 Hz, 8H, $H_{27,30,54,57}$), 3.68 (s, 8H, $H_{28,29,55,56}$), 3.64 (t, *J* = 2.3 Hz, 1H, H_{81}), 1.78 (quintet, *J* = 7.1 Hz, 2H, H_3), 1.73 – 1.67 (m, 18H, $H_{12,19,39,46,66,73}$), 1.50 (sextet, *J* = 7.5 Hz, 2H, H_2), 0.98 (t, *J* = 7.4 Hz, 3H, H_1); **^{13}C NMR** (151 MHz, DMSO) δ 163.30, 156.96, 156.72, 155.43, 149.58,

139.88, 139.77, 139.38, 139.27, 133.81, 133.73, 133.46, 129.79, 129.68, 129.61, 128.93, 128.65, 128.57, 127.46, 127.36, 127.33, 126.14, 126.00, 125.95, 125.22, 124.50, 119.40, 119.33, 119.15, 107.86, 106.98, 106.87, 79.65, 78.82, 70.44, 69.40, 67.63, 67.60, 55.93, 48.57, 31.21, 31.18, 22.15, 22.14, 19.27, 14.21. **HRMS** (ESI$^-$): Calc. for $C_{112}H_{110}N_9O_{16}^-$: 1837.8110, found 1837.8141 [M-H]$^-$. *Due to high degree of apparent symmetry within **L2** many ^{13}C signals overlap.

S3.2 Synthesis of Λ-**L2**•Lu

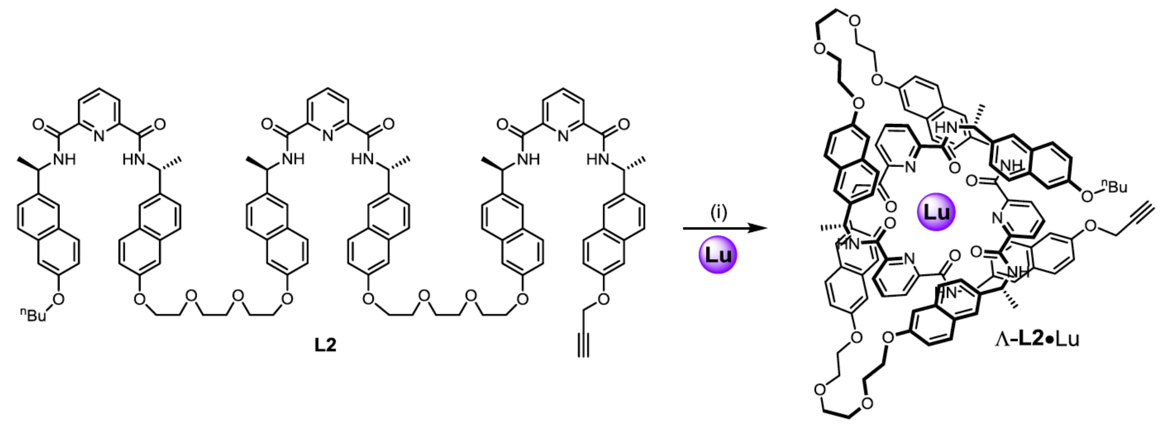

Scheme S2: Synthesis of Λ-**L2**•Lu. Reagents and conditions: (i) $Lu(SO_3CF_3)_3$, MeCN, 80 °C, 16 h, 75%.

Λ-**L2**•Lu

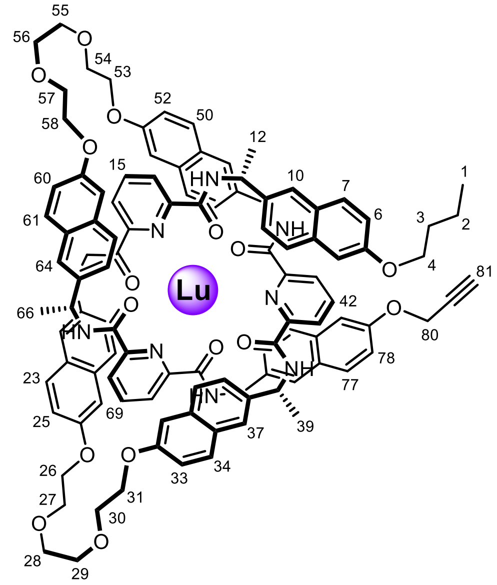

To a stirring solution of **L2** (0.310 g, 0.17 mmol) in acetonitrile (170 mL) was added a solution of lutetium trifluoromethanesulfonate (0.105 g, 0.17 mmol). The reaction was stirred for 16 h at 80 °C. The mixture was allowed to cool to room temperature and concentrated under reduced pressure. The solid was washed with dichloromethane and filtered to give Λ-**L2**•Lu (0.314 g, 75%) as an off-white powder. **^{1}H NMR** (600 MHz, MeCN-d_3) δ 8.44 (d, J = 4.0 Hz, 2H, $H_{40,44}$), 8.40 (dd, J = 12.7, 6.5 Hz, 2H, $H_{13/67,17/71}$), 8.31 (t, J = 4.8 Hz, 2H, $H_{13/67,17/71}$), 7.68 – 7.46 (m, 12H, $H_{7,23,34,50,61,77,8,22,62,76,41,43}$), 7.37 – 7.18 (m, 12H, $H_{35,49,5,24,59,79,6,25,60,78,37,47}$), 7.16 – 7.09 (m, 3H, $H_{33,52,42}$), 7.07 (dd, J = 11.8, 2.1 Hz, 2H, $H_{32,51}$), 7.00 (d, J = 6.2 Hz, 2H, $H_{10/64,20/74}$), 6.96 – 6.89 (m, 4H, $H_{14/68,16/70,10/64,20/74}$), 6.86 – 6.74 (m, 6H, $H_{14/68,16/70,9,21,63,75}$), 6.68 (dd, J = 8.4, 1.4 Hz, 1H, $H_{36/48}$), 6.65 (dd, J = 8.4, 1.4 Hz, 1H, $H_{36/48}$), 6.04 (t, J = 8.0 Hz, 2H, $H_{15,69}$),

4.98 (d, J = 2.1 Hz, 2H, H_{80}), 4.80 – 4.58 (m, 6H, $H_{38,45,11,18,65,72}$), 4.40 – 4.24 (m, 8H, $H_{26,31,53,58}$), 4.21 (t, J = 7.0 Hz, 2H, H_4), 4.00 – 3.78 (m, 16H, $H_{27,28,29,30,54,55,56,57}$), 3.02 (t, J = 2.2 Hz, 1H, H_{81}), 1.93 – 1.87 (m, 2H, H_3), 1.65 – 1.36 (m, 20H, $H_{2,12,19,39,46,66,73}$), 1.08 (t, J = 7.3 Hz, 3H, H_1); **^{13}C NMR** (151 MHz, CD_3CN) δ 166.63, 166.55, 166.37, 166.29, 166.18, 166.08, 157.35, 157.11, 156.94, 156.92, 155.68, 144.37, 144.36, 143.71, 143.67, 143.65, 143.63, 143.62, 140.80, 139.06, 139.05, 138.56, 138.42, 138.40, 137.78, 133.71, 133.37, 133.27, 133.09, 133.06, 129.32, 129.08, 128.86, 128.82, 128.63, 128.36, 128.25, 128.12, 128.08, 127.33, 127.19, 127.13, 126.97, 126.95, 124.22, 124.11, 123.56, 123.52, 123.16, 123.13, 123.07, 123.02, 122.98, 122.44, 122.36, 122.34, 122.27, 122.18, 120.06, 119.52, 119.48, 119.40, 119.37, 119.28, 107.60, 107.07, 107.03, 106.82, 78.77, 76.33, 70.69, 70.60, 69.02, 68.36, 68.21, 68.19, 67.90, 55.77, 53.21, 53.19, 52.73, 52.62, 52.60, 52.13, 51.90, 31.14, 29.91, 22.57, 22.51, 22.38, 21.31, 21.10, 19.14, 13.28. **HRMS** (ESI^+): Calcd. for $C_{112}H_{111}N_9O_{16}Lu$ $[M\text{-}3OTf]^{3+}$: 671.2527, found 671.2513. *Due to the pseudo-symmetry within Λ-**L2**•Lu many ^{13}C signals overlap.

S3.3 Synthesis of **L3**H^+

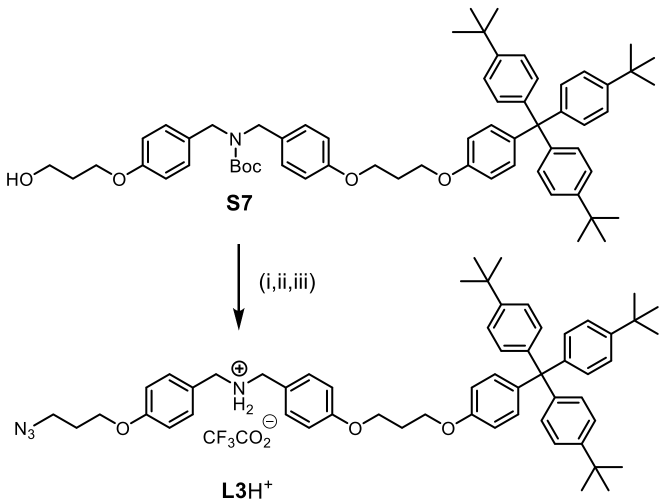

Scheme S3: Synthesis of **L3**H^+. Reagents and conditions: (i) MsCl, Et_3N, CH_2Cl_2, 0 °C, 1 h, 97%. (ii) NaN_3, DMF, 50 °C, 5 h, quant. (iii) CF_3CO_2H, CH_2Cl_2, r.t., 1 h, quant.

S8

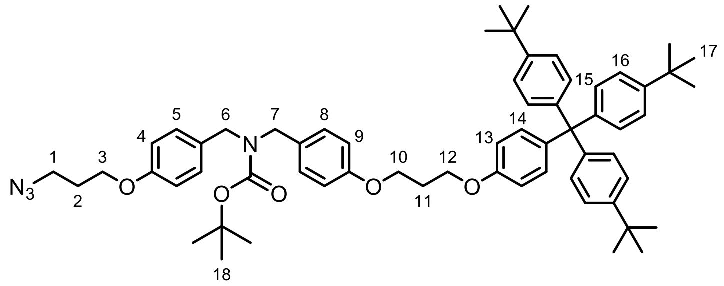

To a solution of **S7**[2] (0.200 g, 0.215 mmol) in CH_2Cl_2 (20 mL) at 0 °C was added triethylamine (0.036 mL, 0.258 mmol) and methanesulfonyl chloride (0.020 mL, 0.258 mmol). The reaction mixture was stirred for 1 hour at 0 °C. The mixture was diluted with CH_2Cl_2 (30 mL), washed with water, dried (Na_2SO_4) and the solvent removed under reduced pressure. The resulting colorless solid (0.210 g, 97%) was used without further purification.

To a solution of the mesylate (0.155 g, 0.15 mmol) in DMF was added sodium azide (0.100 g, 1.53 mmol). The mixture was heated at 50 °C for 5 hours. The mixture was cooled to room temperature, water was added and extracted three times with CH_2Cl_2. The organic washes were combined, dried (Na_2SO_4) and the solvent removed under reduced pressure. The resulting colorless solid was used without further purification (assumed quant.). **^{1}H NMR** (600 MHz, $CDCl_3$) δ 7.22 (d, J = 8.5 Hz, 6H, H_{16}), 7.18 – 7.05 (m, 12H, $H_{5,8,14,15}$), 6.87 – 6.82 (m, 4H, $H_{4,9}$), 6.78 (d, J = 8.9 Hz, 2H, H_{13}), 4.31 (brs, 2H, $H_{6/7}$), 4.23 (brs, 2H, $H_{6/7}$), 4.16 – 4.11 (m, 4H, $H_{10,12}$), 4.04 (t, J = 5.9 Hz, 2H, H_3), 3.52 (t, J = 6.5 Hz, 2H, H_1), 2.24 (quintet, J = 5.9 Hz, 2H, H_{11}), 2.05 (quintet, J = 6.3 Hz, 2H, H_2), 1.49 (s, 9H, H_{18}), 1.29 (s, 27H, H_{17}); **^{13}C NMR** (151 MHz, $CDCl_3$) δ 158.13, 157.89, 156.62, 155.94, 148.29, 144.12, 139.66, 132.26, 130.71, 129.42, 128.77, 124.04, 114.45, 112.93, 79.92, 64.49, 64.17, 63.03, 48.26, 34.30,

31.39, 29.35, 28.82, 28.51. **HRMS** (ES^+): Calcd. for $C_{62}H_{76}N_4O_5K^+$: 995.5447, found 995.5449 $[M+K]^+$.

L3H^+

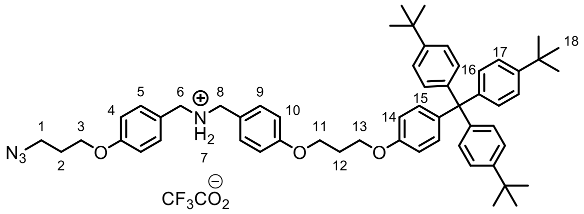

To a solution of **S8** (0.140 g, 0.150 mmol) in CH_2Cl_2 (3 mL) at room temperature was added trifluoroacetic acid (20%, 0.6 mL). The reaction mixture was stirred for 1 hour and concentrated under reduced pressure. The crude residue was triturated with Et_2O. The resulting pale yellow solid was used without further purification (0.140 g, assumed quant.). **^{1}H NMR** (600 MHz, CD_2Cl_2) δ 9.39 (s, 2H, H_7), 7.27 – 7.23 (m, 10H, $H_{5,9,17}$), 7.18 – 7.10 (m, 8H, $H_{15,16}$), 6.88 – 6.84 (m, 4H, $H_{4,10}$), 6.75 (d, *J* = 7.2 Hz, 2H, H_{14}), 4.09 – 4.04 (m, 4H, $H_{11,13}$), 3.95 (t, *J* = 6.0 Hz, 2H, H_3), 3.84 (s, 4H, $H_{6,8}$), 3.45 (t, *J* = 5.5 Hz, 2H, H_1) 2.17 (quintet, *J* = 6.0 Hz, 2H, H_{12}), 2.01 – 1.95 (m, 2H, H_2), 1.29 – 1.28 (m, 27H, H_{18}); **HRMS** (ES^+): Calc. for $C_{57}H_{68}N_4O_3K^+$: 895.4923, found 895.4892 $[M+K]^+$.

S3.4 Synthesis of **1**H$^+$

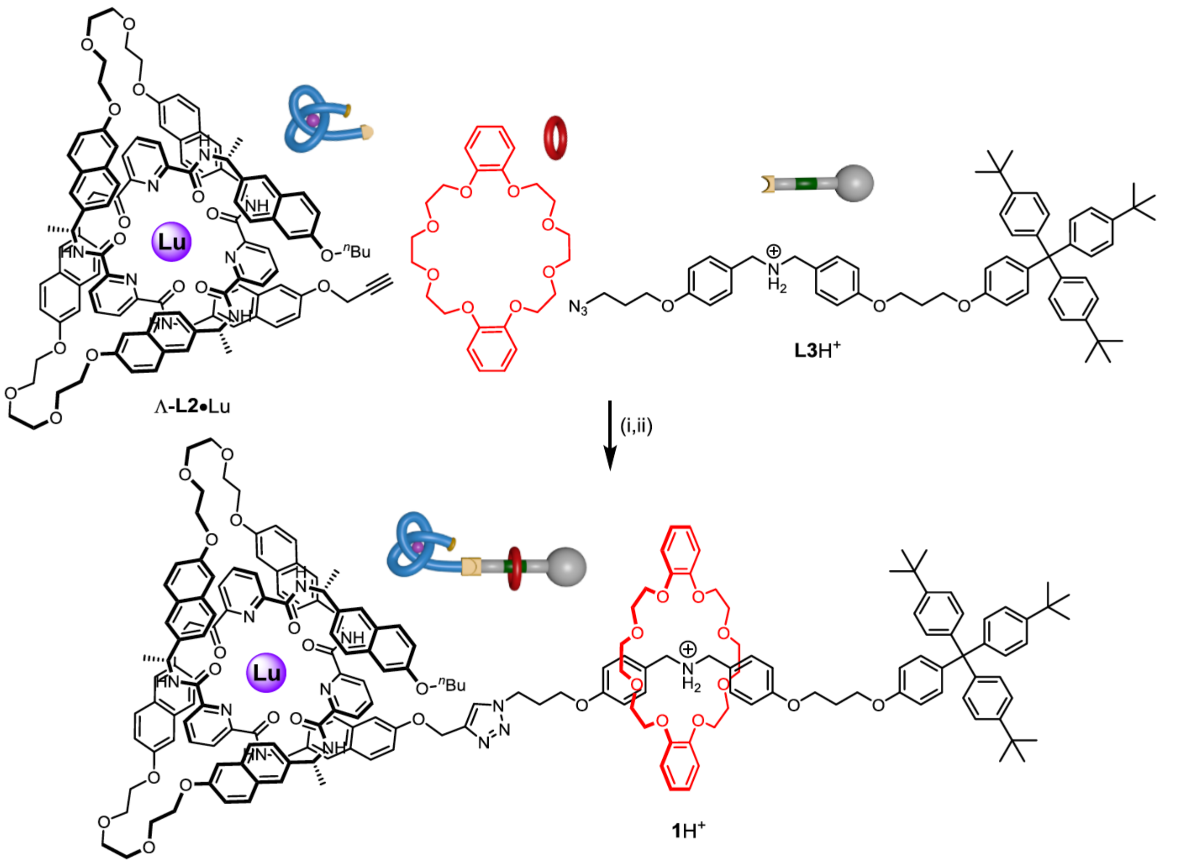

Scheme S4: Synthesis of **1**H$^+$. Reagents and conditions: (i) DB24C8, CF_3CO_2H, CD_2Cl_2, r.t., 10 min. (ii) Λ-**L2**•Lu, $Cu(MeCN)_4\cdot CF_3SO_3$, MeOH:MeCN (1:1), r.t., 20 h, 41%.

1H$^+$

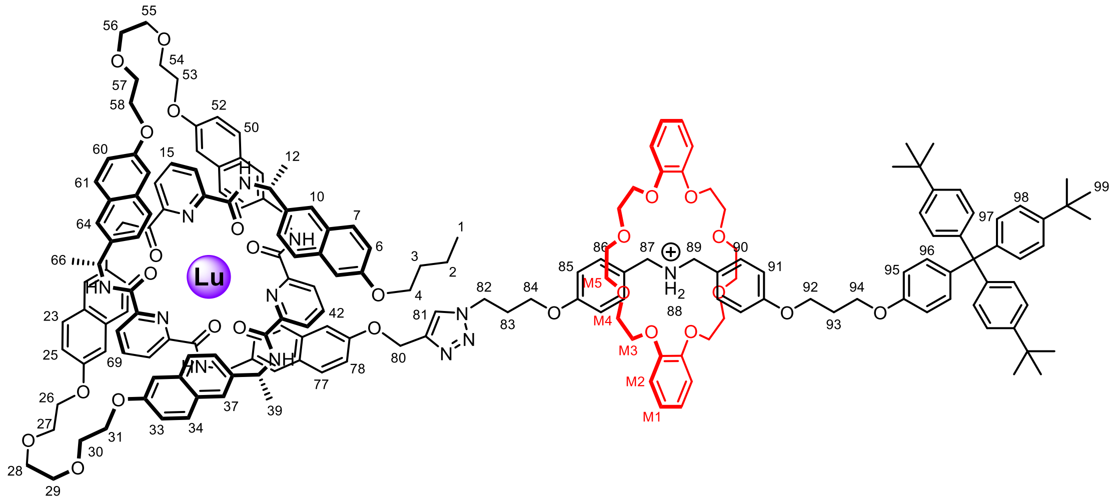

Due to the evaporation of some trifluoroacetic acid during storage of **L3**H$^+$ under vacuum, the molecule was always freshly treated with trifluoroacetic acid to ensure full protonation before threading. To a solution of **L3**H$^+$ (12.1 mg, 0.0125 mmol) in CD_2Cl_2 (0.5 mL 0.025M) was added trifluoroacetic acid (10.0 µL, 0.125 mmol). To this, DB24C8 (22.4 mg, 0.050 mmol) was added and the mixture was left to stir for 10 minutes. The mixture was azeotroped three times with toluene (to remove excess TFA), evaporated under reduced pressure and dissolved into a degassed 1:1 mixture of MeOH:MeCN (1.25 mL, 0.01 M). Λ-**L2**•Lu (15.4 mg, 0.00625 mmol) and $Cu(MeCN)_4{\cdot}CF_3SO_3$ (9.4 mg, 0.025 mmol) were added to the reaction mixture and stirred at room temperature for 20 hours. The solvent was removed under reduced pressure. The resulting solid was sonicated in toluene and filtered (to remove excess DB24C8). The solid was redissolved in CH_2Cl_2, swiftly washed with saturated aqueous EDTA solution, dried with $MgSO_4$ and evaporated under reduced pressure. Purification by size exclusion chromatography (SX-1 beads, CH_2Cl_2 eluent) gave the title compound **1**H$^+$ as a colorless solid (10.1 mg, 41%). **^{1}H NMR** (600 MHz, MeCN-d_3) δ 8.57 – 8.47 (m, 3H, $H_{40,44,13/17/67/71}$), 8.44 (d, J = 6.3 Hz, 1H, $H_{13/17/67/71}$), 8.35 – 8.27 (m, 2H, $H_{13/17/67/71}$), 7.79 – 7.43 (m, 12H, $H_{7,23,34,50,61,77,8,22,62,76,41,43}$), 7.39 – 7.16 (m, 27H, $H_{35,49,5,24,59,79,6,25,60,78,37,47,88,96,98,81,86,90}$), 7.16 – 7.09 (m, 9H, $H_{33,52,42,97}$), 7.09 – 7.05 (m, 2H, $H_{32,51}$), 7.00 (d, J = 6.4 Hz, 2H, $H_{10/64,20/74}$), 6.95 – 6.72 (m, 20H, $H_{9,14,16,21,63,68,70,75,95,10/64,20/74,M1,M2}$), 6.68 – 6.59 (m, 6H, $H_{48,36,85,91}$), 6.03 (t, J = 7.8 Hz, 2H, $H_{15,69}$), 5.33 (m, 2H, H_{80}), 4.81 – 4.72 (m, 2H, $H_{38,45}$), 4.71 – 4.59 (m, 6H, $H_{82,11,18,65,72}$), 4.58 – 4.49 (m, 4H, $H_{87,89}$), 4.35 – 4.22 (m, 8H, $H_{26,31,53,58}$), 4.18 (t, J = 6.7 Hz, 2H, H_4), 4.13 – 3.77 (m, 30H, $H_{27,28,29,30,54,55,56,57,84,92,94,M3}$), 3.76 – 3.68 (m, 8H, H_{M4}), 3.59 – 3.51 (m, 8H, H_{M5}), 2.37 – 2.31 (m, 2H, H_{83}), 2.15 – 2.10 (m, 2H, H_{93}), 1.89 – 1.81 (m, 2H, H_3), 1.64 – 1.40 (m, 20H, $H_{2,12,19,39,46,66,73}$), 1.26 (s, 27H, H_{99}), 1.02 (t, J = 7.5 Hz, 3H, H_1); **^{13}C NMR** (151 MHz, CD_3CN)

δ 166.71, 166.39, 166.20, 166.11, 159.26, 158.99, 157.40, 157.12, 156.92, 156.80, 156.65, 148.48, 147.39, 144.57, 144.54, 144.46, 143.73, 143.64, 140.93, 139.66, 138.98, 138.50, 138.44, 138.41, 137.87, 133.79, 133.56, 133.41, 133.09, 131.71, 130.88, 130.84, 130.20, 129.31, 129.16, 129.12, 128.84, 128.56, 128.38, 128.31, 128.11, 127.33, 127.23, 126.97, 124.46, 124.02, 123.62, 123.31, 123.25, 123.19, 122.49, 122.42, 122.35, 122.20, 121.14, 120.07, 119.54, 119.40, 114.23, 113.33, 112.32, 107.53, 107.12, 107.07, 106.87, 70.70, 70.62, 70.59, 70.14, 69.02, 68.37, 68.20, 67.85, 67.80, 64.56, 64.27, 64.20, 62.99, 61.67, 54.35, 53.16, 52.67, 51.96, 51.85, 51.75, 51.67, 47.13, 33.95, 31.13, 30.56, 29.91, 29.68, 29.17, 28.82, 22.57, 22.43, 22.39, 21.15, 21.03, 19.89, 19.10, 13.28. **HRMS** (ESI^+): Calcd. for $C_{193}H_{212}N_{13}O_{27}Lu$ $[M\text{-}3OTf\text{-}OCOCF_3]^{4+}$: 830.1267, found 830.1230. *Due to high degree of apparent symmetry within **1**H^+ many ^{13}C signals overlap.

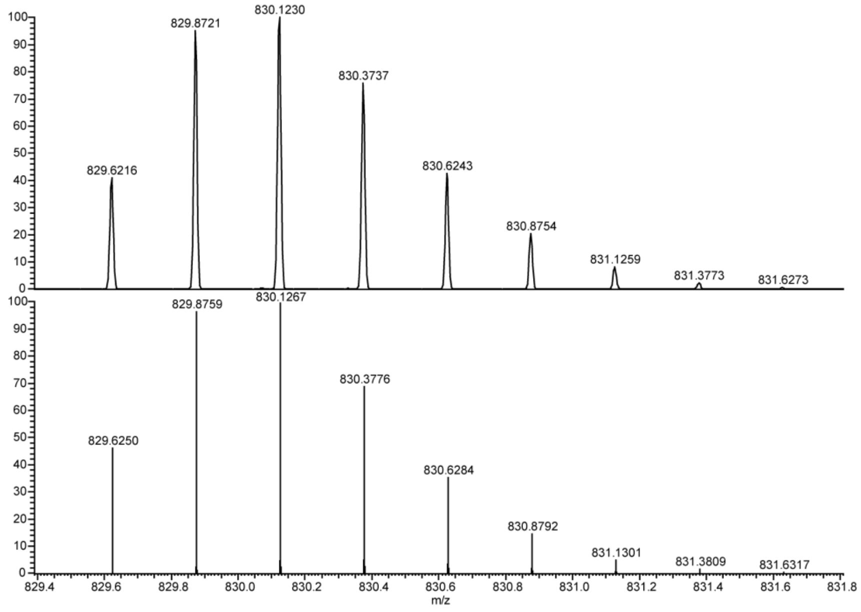

Figure S1. Isotopic distribution of **1**H^+ $[M]^{4+}$, observed (top) and predicted (bottom).

S3.5 Synthesis of **1**

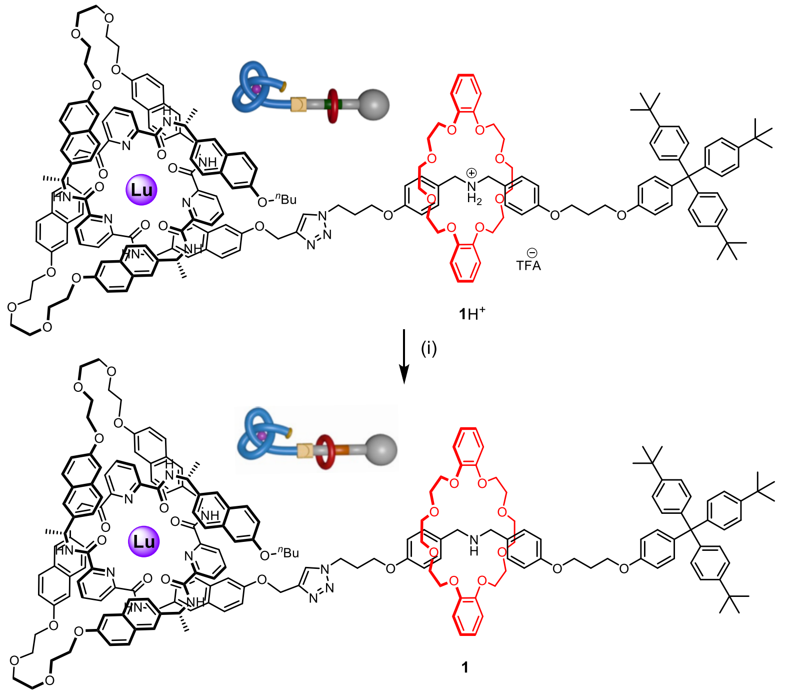

Scheme S5: Synthesis of **1**. Reagents and conditions: (i) Et_3N (10 equiv.), CD_2Cl_2, r.t., 1 hour.

1

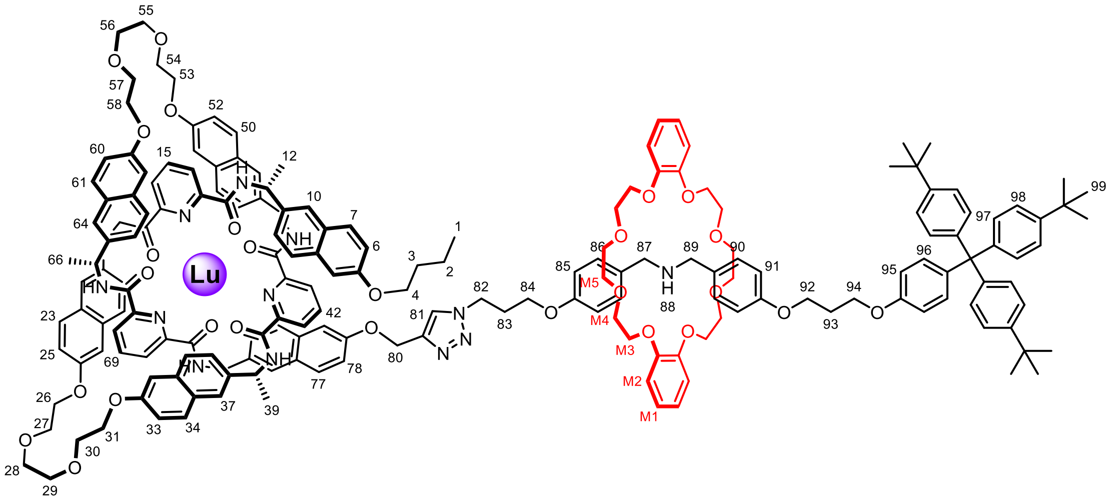

To a solution of **1**H$^+$ (3.6 mg, 0.931 μmol) in CD_2Cl_2 (0.6 mL) in an NMR tube was added Et_3N (1.29 μL, 9.3 μmol). The mixture was monitored by ^{1}H NMR and complete deprotonation was established after 1 hour. **^{1}H NMR** (600 MHz, MeCN-*d*$_3$) *δ* 8.58 (d, *J* = 4.3 Hz, 1H, $H_{40/44}$), *δ* 8.52 (d, *J* = 4.3 Hz, 1H, $H_{40/44}$), 8.48 (d, *J* = 6.5 Hz, 1H, $H_{13/17/67/71}$), 8.42 (d, *J* = 6.0 Hz, 1H, $H_{13/17/67/71}$), 8.32 – 8.26 (m, 2H, $H_{13/17/67/71}$), 8.05 (s, 1H, H_{81}), 7.68 – 7.46 (m, 12H, $H_{7,23,34,50,61,77,8,22,62,76,41,43}$), 7.39 – 7.17 (m, 25H, $H_{35,49,5,24,59,79,6,25,60,78,37,47,88,96,98,86,90}$), 7.16 – 7.09 (m, 9H, $H_{33,52,42,97}$), 7.07 (dd, *J* = 5.2, 2.2 Hz, 2H, $H_{32,51}$), 7.00 (d, *J* = 5.5 Hz, 2H, $H_{10/64,20/74}$), 6.95 – 6.72 (m, 20H, $H_{9,14,16,21,63,68,70,75,95,10/64,20/74,M1,M2}$), 6.68 – 6.60 (m, 6H,

$H_{48,36,85,91}$), 6.03 (t, J = 8.0 Hz, 2H, $H_{15,69}$), 5.38 (d, J = 4.8 Hz, 2H, H_{80}), 4.81 – 4.72 (m, 2H, $H_{38,45}$), 4.71 – 4.62 (m, 4H, $H_{11,18,65,72}$), 4.61 – 4.49 (m, 6H, $H_{82,87,89}$), 4.37 – 4.22 (m, 8H, $H_{26,31,53,58}$), 4.17 (t, J = 6.5 Hz, 2H, H_4), 4.12 – 3.78 (m, 30H, $H_{27,28,29,30,54,55,56,57,84,92,94,M3}$), 3.76 – 3.68 (m, 8H, H_{M4}), 3.59 – 3.51 (m, 8H, H_{M5}), 2.33 (quintet, J = 6.8 Hz, 2H, H_{83}), 2.15 – 2.10 (m, 2H, H_{93}), 1.88 – 1.80 (m, 2H, H_3), 1.64 – 1.42 (m, 20H, $H_{2,12,19,39,46,66,73}$), 1.26 (s, 27H, H_{99}), 1.02 (t, J = 7.5 Hz, 3H, H_1); **^{13}C NMR** (151 MHz, MeCN-d_3) δ 167.61, 167.29, 167.09, 167.01, 160.17, 159.90, 158.31, 158.04, 157.83, 157.71, 157.57, 149.39, 148.30, 145.45, 145.36, 144.63, 144.55, 144.18, 140.57, 139.91, 139.39, 139.34, 139.30, 138.76, 134.70, 134.46, 134.32, 134.31, 134.00, 132.62, 131.79, 131.75, 130.21, 130.07, 130.02, 129.75, 129.46, 129.29, 129.21, 129.02, 129.01, 128.23, 128.14, 128.11, 127.88, 125.70, 125.38, 125.32, 125.26, 125.24, 125.20, 124.93, 124.56, 124.51, 124.21, 124.15, 124.07, 123.91, 123.89, 123.38, 123.34, 123.25, 123.12, 123.07, 123.06, 122.05, 120.99, 120.45, 120.41, 120.36, 120.31, 120.30, 115.16, 115.14, 114.24, 113.23, 108.42, 108.02, 107.97, 107.76, 79.11, 78.89, 78.68, 71.62, 71.54, 71.51, 71.05, 69.94, 69.29, 69.12, 68.77, 68.72, 65.47, 65.19, 65.12, 63.91, 62.60, 54.12, 54.08, 53.60, 52.88, 52.77, 52.67, 52.58, 48.04, 34.87, 32.04, 31.49, 30.83, 30.61, 29.74, 23.49, 23.33, 23.30, 22.08, 21.96, 20.02, 14.20, 0.65. **HRMS** (ESI$^+$): Calcd. for $C_{193}H_{211}N_{13}O_{27}Lu$ $[M\text{-}3OTf]^{3+}$: 1106.4999 found 1106.4953. *Due to high degree of apparent symmetry within **1** many ^{13}C signals overlap.

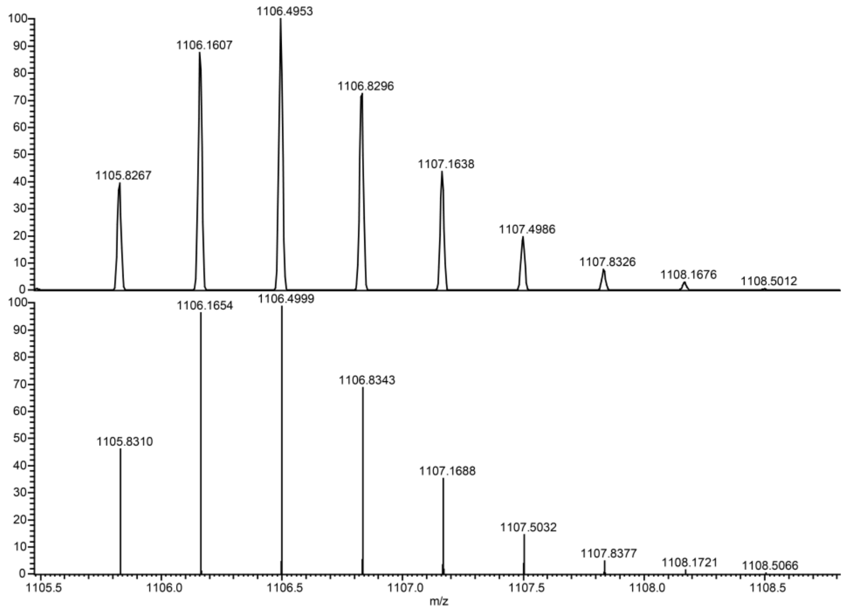

Figure S2. Isotopic distribution of **1** $[M]^{3+}$, observed (top) and predicted (bottom).

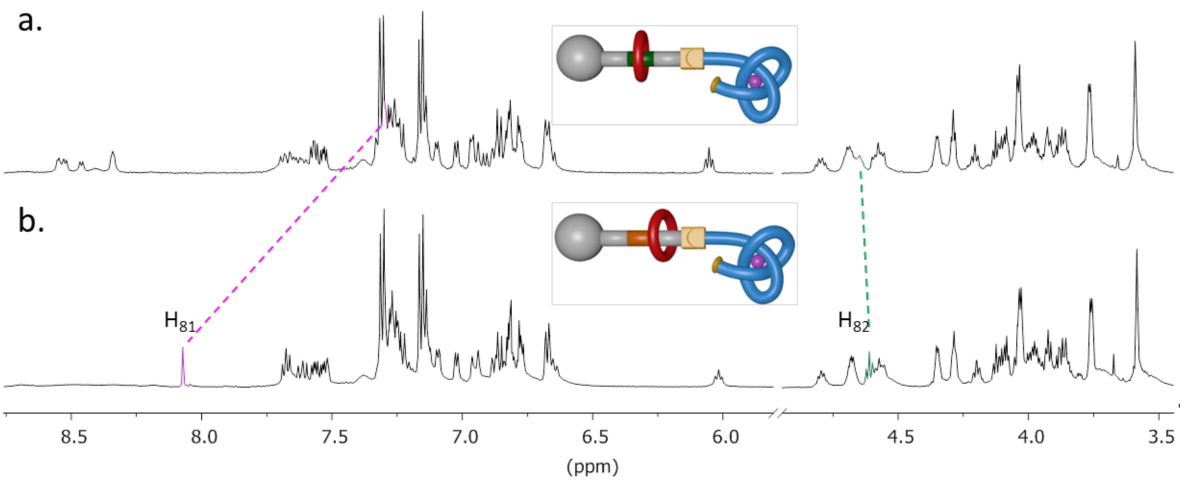

Figure S3. Deprotonation of rotaxane architecture **1**H$^+$. (a) Partial ^{1}H NMR (600 MHz, MeCN-*d*$_3$) of **1**H$^+$. (b) Partial ^{1}H NMR (600 MHz, MeCN-*d*$_3$) of **1**, recorded *in situ* after addition of NEt_3 (10 equiv.) to **1**H$^+$. Notice that the amide *N-H* signals around 8.5 ppm disappear in the presence of base.

Note: ^{1}H NMR shifts upon deprotonation of **1**H$^+$ indicate that the macrocycle interacts some of the time with the triazole ring in the deprotonated state **1**, as evidenced by shifts of protons H_{81} and H_{82}. However, the benzylic protons H_{87}/H_{89} only shift to a modest extent. This indicates that the macrocycle is not localized to one particular region after deprotonation, but samples different regions of the track.

S3.6 Synthesis of Λ-**L1**H$^+$•Lu

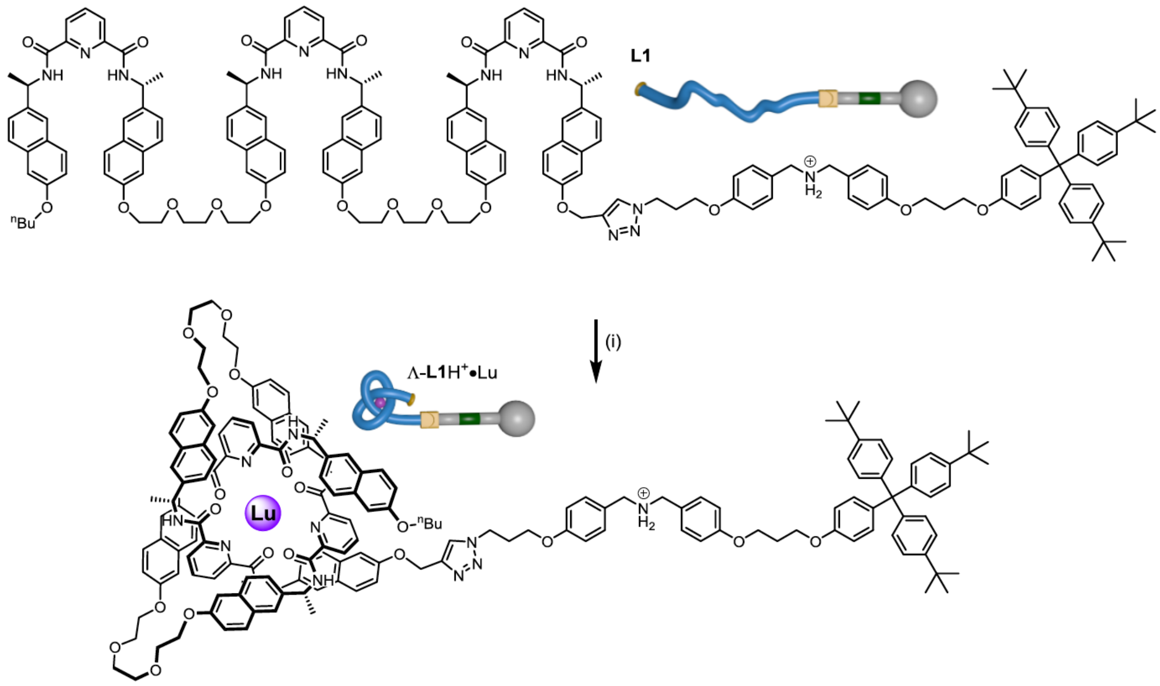

Scheme S6: Synthesis of Λ-**L1**H$^+$•Lu. Reagents and conditions: (i) $Lu(SO_3CF_3)_3$, MeCN, 80 °C, 20 h, 58 %.

Λ-**L1**H$^+$•Lu

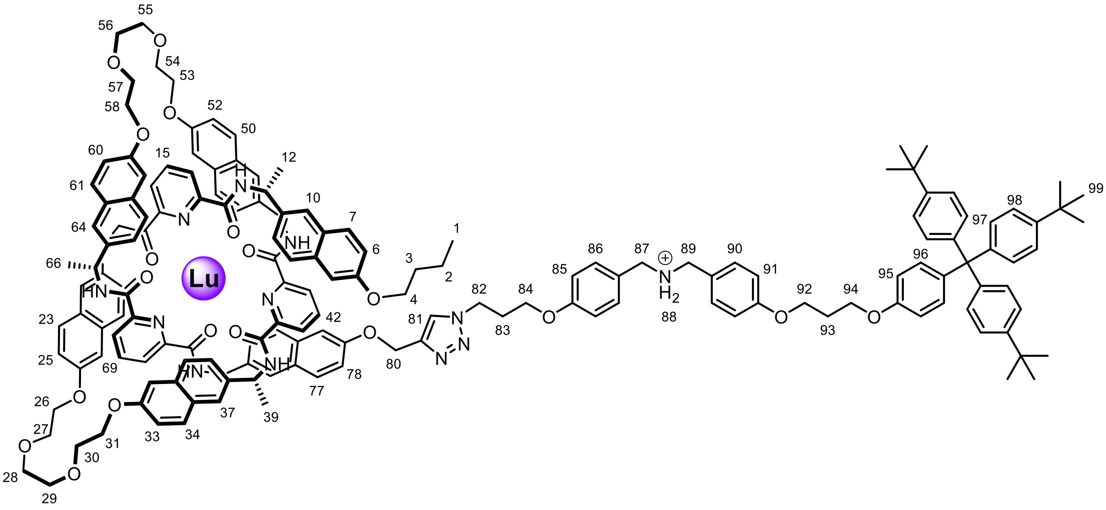

To a solution of **L1** (7.0 mg, 2.6 µmol) in MeCN (2.6 mL) was added lutetium trifluoromethanesulfonate (3.2 mg, 5.1 µmol) and stirred at 80 °C for 20 hours. The solvent was removed under reduced pressure. The crude was washed with toluene (2 x 5 mL) to yield the title compound as a colorless solid (5.0 mg, 58 %). **^{1}H NMR** (600 MHz, MeCN-d_3) δ **^{1}H NMR** (600 MHz, MeCN-d_3) δ 8.55 (d, J = 4.5 Hz, 1H, $H_{40/44}$), 8.52 (d, J = 4.6 Hz, 1H,

$H_{40/44}$), 8.50 (d, *J* = 6.5 Hz, 1H, $H_{13/17/67/71}$), 8.48 – 8.43 (m, 1H, $H_{13/17/67/71}$), 8.35 – 8.32 (m, 2H, $H_{13/17/67/71}$), 8.04 (s, 1H, H_{81}), 7.68 – 7.48 (m, 12H, $H_{7,8,22,23,34,41,43,50,61,62,76,77}$), 7.38 – 7.35 (m, 4H, $H_{86,90}$), 7.31 – 7.17 (m, 20H, $H_{5,6,24,25,35,37,47,49,59,60,78,79,96,98}$), 7.16 – 7.10 (m, 9H, $H_{33,42,52,97}$), 7.07 (m, 4H, $H_{32,51,88}$), 7.00 – 6.98 (m, 2H, $H_{10/64,20/74}$), 6.97 – 6.92 (m, 8H, $H_{10/64,20/74,14/16/68/70,14/16/68/70,85,91}$), 6.89 (d, *J* = 8.0 Hz, 1H, $H_{14/16/68/70}$), 6.87 – 6.77 (m, 7H, $H_{14/16/68/70,9,21,63,75,95}$), 6.68 – 6.61 (m, 2H, $H_{36,48}$), 6.00 (t, *J* = 7.9 Hz, 2H, $H_{15,69}$), 5.40 – 5.37 (m, 2H, H_{80}), 4.79 – 4.74 (m, 2H, $H_{38,45}$), 4.68 – 4.63 (m, 4H, $H_{11,18,65,72}$), 4.60 (t, *J* = 6.9 Hz, 2H, H_{82}), 4.32 – 4.23 (m, 8H, $H_{26,31,53,58}$), 4.17 (dt, *J* = 6.5, 1.7 Hz, 2H, H_4), 4.15 – 4.05 (m, 12H, $H_{27/30/54/57,87,89,92,94}$), 4.01 (t, *J* = 5.8 Hz, 2H, H_{84}), 3.97 – 3.80 (m, 12H, $H_{27/30/54/57,28,29,55,56}$), 2.36 (quint, *J* = 6.2 Hz, 2H, H_{83}), 2.18 (quint, *J* = 6.2 Hz, 2H, H_{93}), 1.85 (quint, *J* = 6.7 Hz, 2H, H_3), 1.60 – 1.44 (m, 20H, $H_{2,12,19,39,46,66,73}$), 1.27 (s, 27H, H_{99}), 1.02 (t, *J* = 7.4 H, 3H, H_1); **^{13}C NMR** (151 MHz, MeCN-*d_3*) δ 166.70, 166.38, 166.19, 166.10, 159.93, 159.66, 157.40, 157.12, 156.92, 156.74, 156.63, 148.47, 144.58, 144.45, 143.72, 143.63, 143.27, 140.96, 139.67, 138.99, 138.47, 138.43, 138.39, 137.83, 133.78, 133.54, 133.41, 133.09, 131.87, 131.85, 131.68, 130.20, 129.86, 129.74, 129.31, 129.14, 129.10, 128.94, 128.84, 128.55, 128.38, 128.30, 128.24, 128.10, 127.31, 127.23, 127.19, 126.97, 124.49, 124.32, 124.05, 123.61, 123.28, 123.24, 123.22, 123.13, 122.98, 122.96, 122.60, 122.48, 122.44, 122.42, 122.40, 122.34, 122.17, 122.14, 122.12, 121.93, 119.81, 119.54, 119.50, 119.40, 117.35, 114.79, 113.32, 107.06, 106.84, 78.20, 77.98, 77.76, 70.69, 70.59, 69.03, 68.36, 68.19, 67.85, 64.71, 64.57, 64.19, 63.01, 61.64, 53.21, 53.17, 52.67, 51.97, 51.85, 50.72, 50.63, 47.07, 35.01, 33.96, 31.65, 31.12, 29.68, 29.22, 29.06, 29.00, 28.92, 28.84, 28.77, 26.85, 26.82, 25.04, 22.57, 22.41, 22.38, 21.17, 21.02, 19.10, 13.43, 13.28. **HRMS** (ESI^+): Calcd. for $C_{169}H_{180}N_{13}O_{19}Lu$ [M-3OTf-OCOCF$_3$]$^{4+}$: 717.5715, found 717.5726. *Due to high degree of apparent symmetry within Λ-**L1**H^+•Lu many ^{13}C signals overlap.

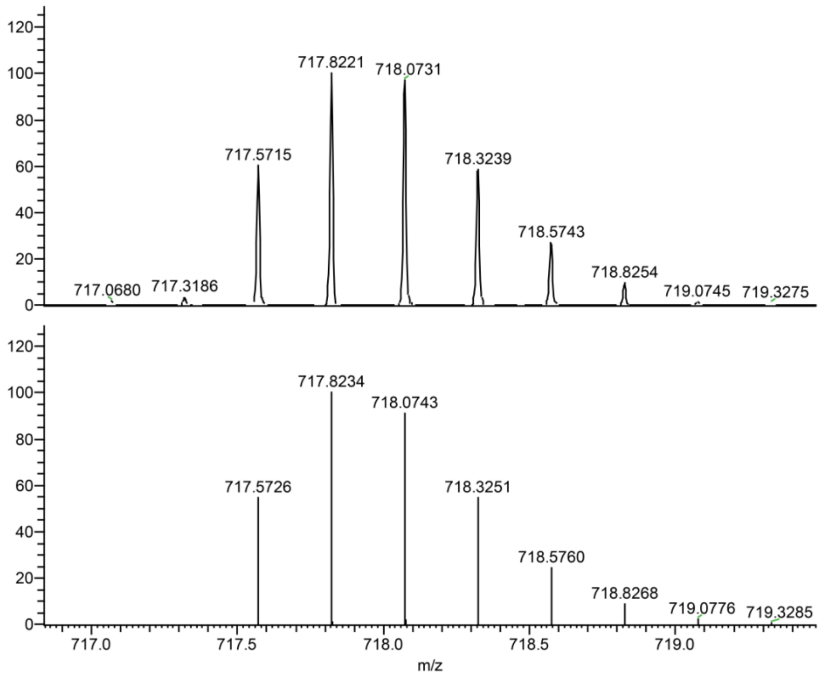

Figure S4. Isotopic distribution of Λ-**L1**H^+•Lu $[M]^{4+}$, observed (top) and predicted (bottom).

S3.7 Dethreading of **1**

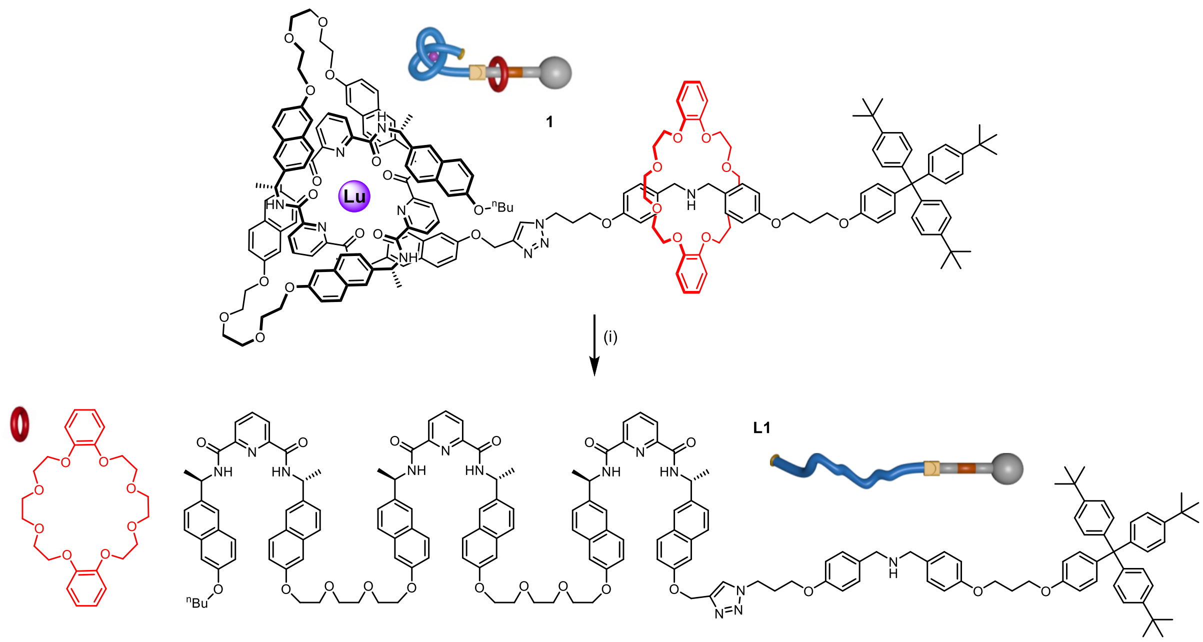

Scheme S7: Demetallation and dethreading of **1**. Reagents and conditions: (i) TEAF, CD_2Cl_2, r.t., 5 minutes.

L1

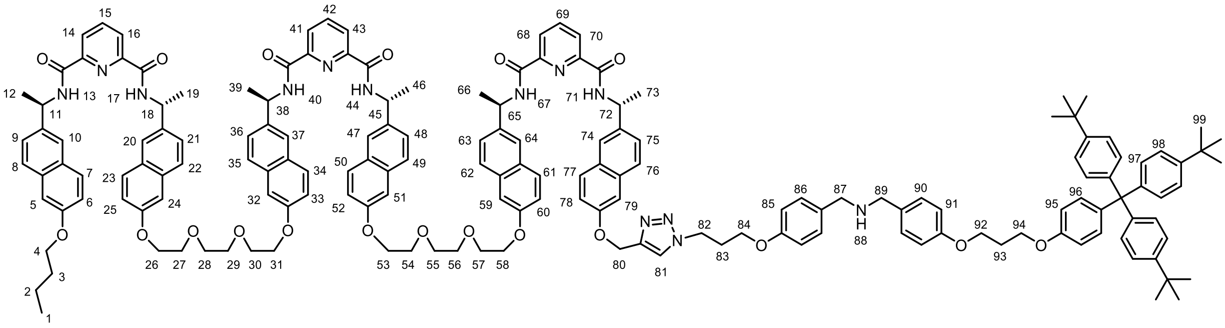

To a solution of **1** (3.6 mg, 0.93 μmol) in CD_2Cl_2 (0.6 mL) was added tetraethylammonium fluoride (1.4 mg, 9.3 μmol). The mixture was left to stir at room temperature for 5 minutes. The mixture was washed with water, dried over $MgSO_4$ and evaporated to dryness. The title compound was obtained as a mixture of the free thread and macrocycle. Purification by trituration with toluene (3 x 1 mL) provided the pure compound. **^{1}H NMR** (600 MHz, CD_2Cl_2) δ 8.29 – 8.24 (m, 6H, $H_{14,16,41,43,68,70}$), 8.10 – 8.02 (m, 6H, $H_{13,17,40,44,67,71}$), 7.98 – 7.93 (m, 3H, $H_{15,42,69}$), 7.72 – 7.60 (m, 18H, $H_{7,8,10,20,22,23,34,35,37,47,49,50,61,62,64,74,76,77}$), 7.44 – 7.38 (m, 7H, $H_{9,21,36,48,63,75,81}$), 7.27 – 7.24 (m, 6H, H_{98}), 7.24 – 7.22 (m, 4H, $H_{86,90}$), 7.17 – 7.15 (m, 8H, $H_{96,97}$), 7.14 – 7.10 (m, 12H, $H_{5,6,24,25,32,33,51,52,59,60,78,79}$), 6.85 (d, J = 8.6 Hz, 2H, $H_{85/91}$), 6.82 (d, J = 8.6 Hz, 2H, $H_{85/91}$), 6.79 (d, J = 8.9 Hz, 2H, H_{95}), 5.36 (m, 6H, $H_{11,18,38,45,65,72}$), 5.26 (s, 2H, H_{80}) 4.55 (t, J = 6.6 Hz, 2H, H_{82}), 4.22 – 4.18 (m, 8H, $H_{26,31,53,58,}$), 4.12 (t, J = 5.4 Hz, 4H, $H_{92,94}$), 4.06 (t, J = 6.5 Hz, 2H, H_4), 3.95 (t, J = 5.7 Hz, 2H, H_{84}), 3.90 – 3.86 (m, 8H, $H_{27,30,54,57}$), 3.74 – 3.73 (m, 8H, $H_{28,29,55,56}$), 3.70 – 3.67 (m, 4H, $H_{87,89}$), 2.36 (quintet, J = 6.1

Hz, 2H, H_{83}); 2.22 (quintet, J = 6.1 Hz, 2H, H_{93}); 1.82 (quintet, J = 7.3 Hz, 2H, H_3), 1.62 (m, 18H, $H_{12,19,39,46,66,73}$); 1.54 (m, 2H, H_2), 1.30 (s, 27H, H_{99}); 1.00 (t, J = 7.4 Hz, 3H, H_1); **^{13}C NMR** (151 MHz, CD_2Cl_2) δ 162.59, 157.86, 157.45, 157.25, 156.86, 156.70, 156.35, 148.97, 148.95, 148.33, 144.48, 143.61, 139.69, 138.88, 138.37, 138.34, 138.15, 133.87, 133.75, 133.34, 132.86, 131.85, 130.35, 129.25, 129.23, 129.17, 129.08, 128.73, 128.72, 127.34, 127.24, 127.16, 125.22, 125.21, 125.13, 125.03, 124.91, 124.88, 124.29, 124.26, 123.39, 119.33, 119.19, 119.13, 114.17, 114.16, 113.12, 106.90, 106.52, 106.26, 77.54, 77.33, 77.12, 70.82, 69.66, 67.75, 67.51, 64.45, 64.35, 64.18, 63.05, 61.90, 48.86, 47.23, 34.17, 31.29, 31.06, 30.61, 29.96, 29.32, 21.40, 21.33, 19.30, 13.67, 1.71, 0.76; **HRMS** (ES^+): Calc. for $C_{169}H_{179}N_{13}O_{19}K_2^{2+}$: 1386.6360, found 1386.6331 $[M+2K]^{2+}$. *Proton signal of H_{88} is not observed. *Due to high degree of apparent symmetry within **L1** many ^{13}C signals overlap.

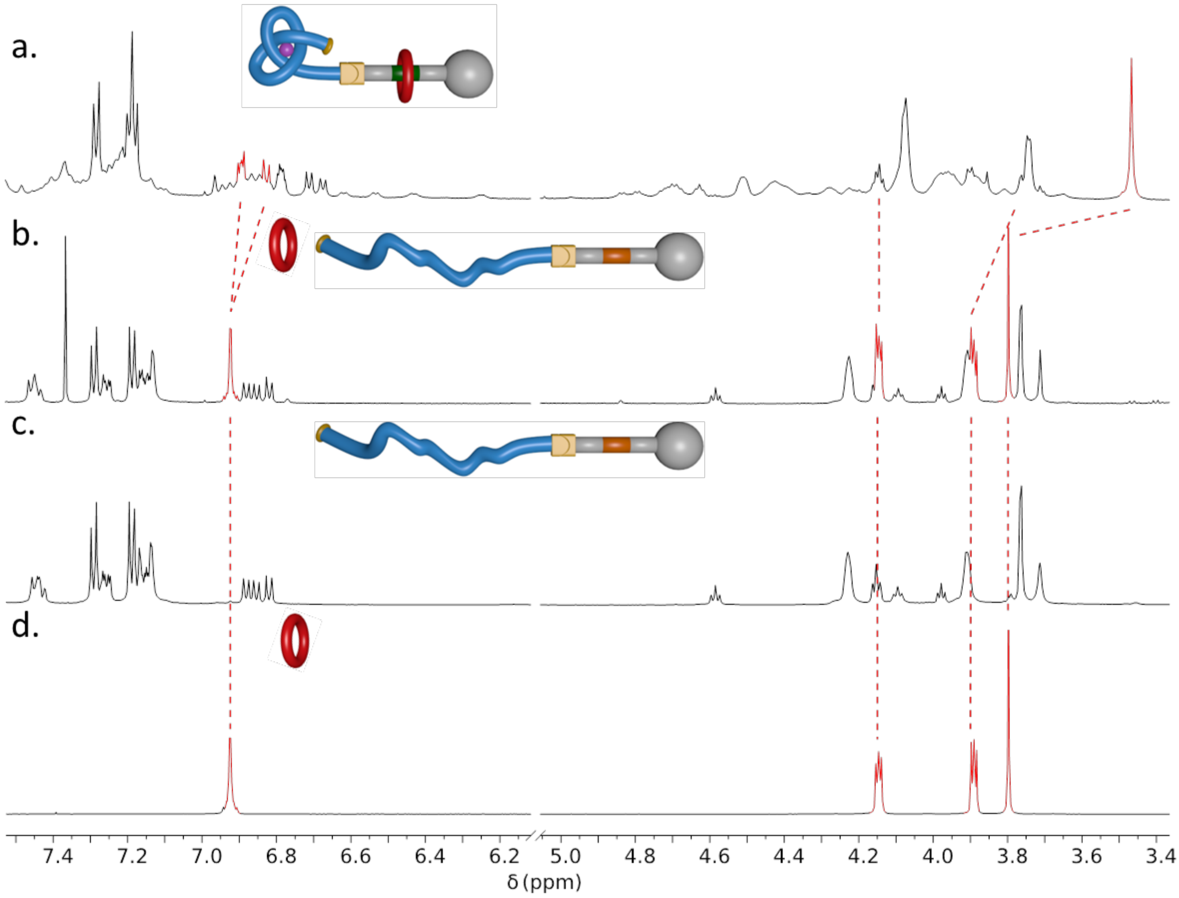

Figure S5. Dethreading of **1**. (a) Partial ^{1}H NMR (600 MHz, CD_2Cl_2) of **1**. (b) Partial ^{1}H NMR (600 MHz, CD_2Cl_2) of crude dethreading reaction mixture. (c) Partial ^{1}H NMR (600 MHz, CD_2Cl_2) of isolated thread **L1**. (d) Partial ^{1}H NMR (600 MHz, CD_2Cl_2) of DB24C8.

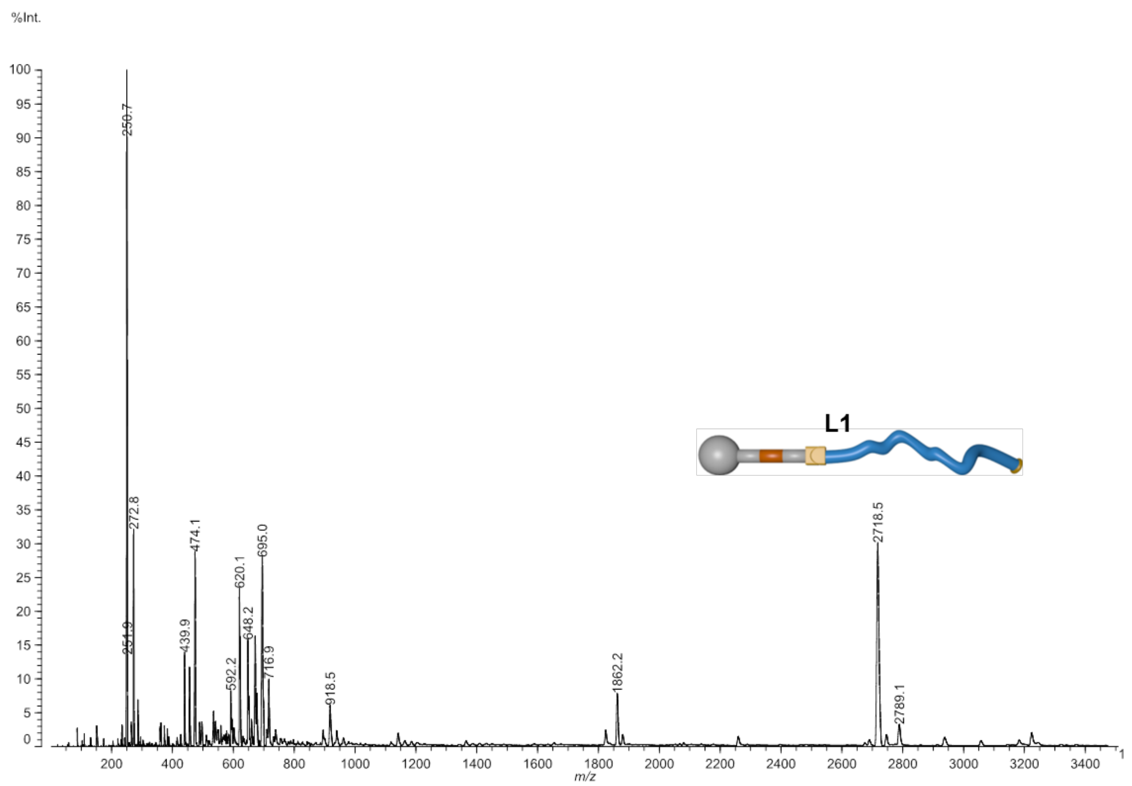

Figure S6. MALDI-MS (positive mode) of the crude reaction mixture after the controlled dethreading described in Scheme S7. Calc. for [$C_{169}H_{179}N_{13}O_{19}Na^{+}$]: 2718.4, found 2718.5.

S3.8 Threading and remetallation of **L1**

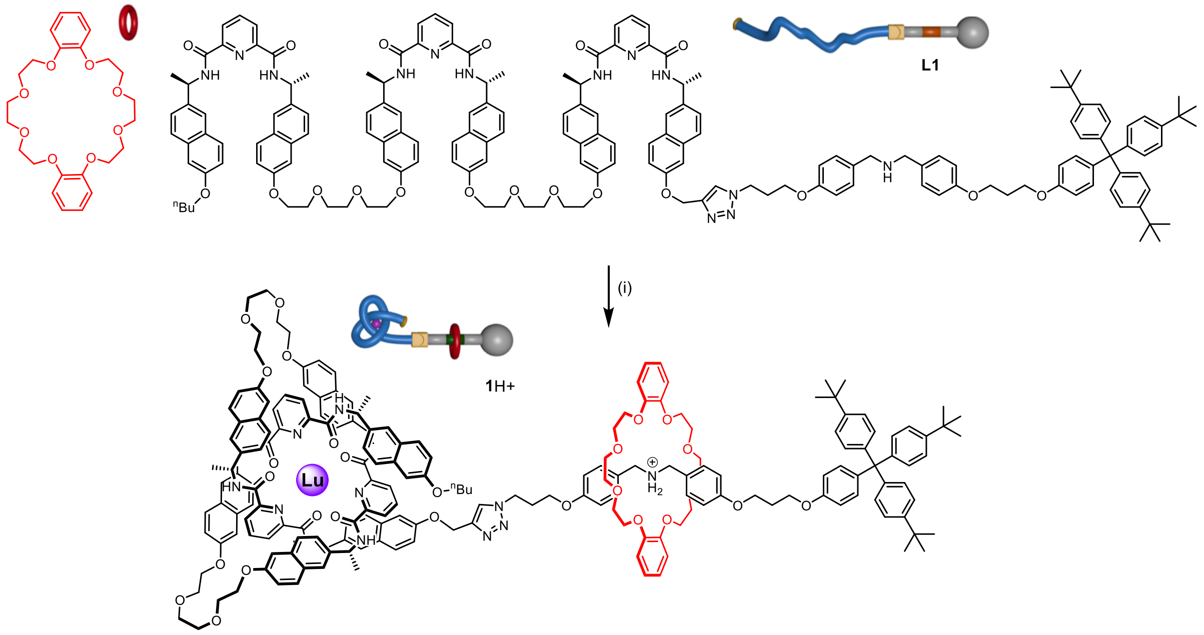

Scheme S8: Threading and remetallation of **L1**. Reagents and conditions: (i) CF_3CO_2H, CD_2Cl_2, r.t., 2 days. (ii) $Lu(SO_3CF_3)_3$, MeCN, 80 °C, 16 h, 90%

To a solution of **L1** (2.1 mg, 0.7 μmol) in CD_2Cl_2 (0.5 ml, 1.5 mM) in an NMR tube was added trifluoroacetic acid (3 μL, 50 equiv.). DB24C8 (3.4 mg, 7 μmol) was added and the threading process was monitored by ^{1}H NMR until full conversion was observed. The mixture was evaporated to dryness and azeotroped three times with toluene. The resulting mixture was suspended in MeCN (0.77 ml, 1 mM) and stirred at 80 °C until all the solids were completely dissolved. To the resulting solution, lutetium trifluoromethanesulfonate (0.96 mg, 1.5 μmol) was added and stirred overnight at 80 °C. The mixture was allowed to cool to room temperature and concentrated under reduced pressure. The degree of rethreading/remetallation was determined by integration of ^{1}H NMR signals (see figures S7 and S8).

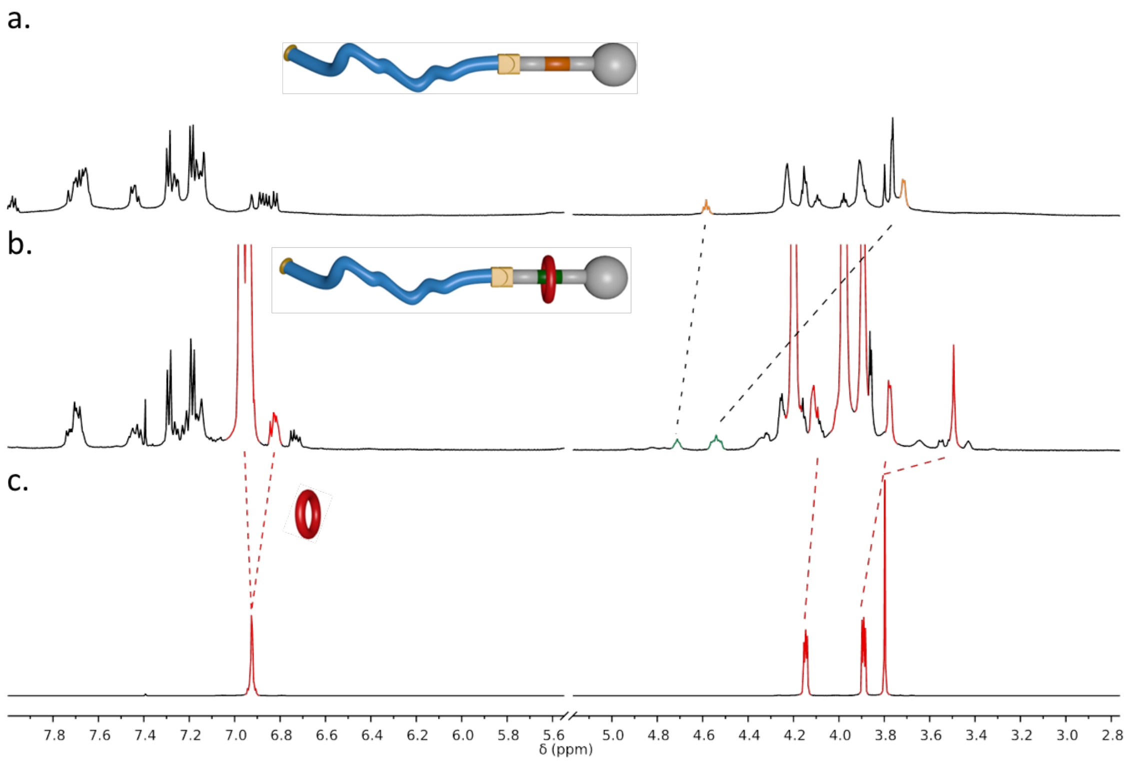

Figure S7. Rethreading of **L1**. (a) Partial ^{1}H NMR (600 MHz, CD_2Cl_2) of **L1**. (b) Partial ^{1}H NMR (600 MHz, CD_2Cl_2) of crude rethreading reaction mixture. (c) Partial ^{1}H NMR (600 MHz, CD_2Cl_2) of DB24C8.

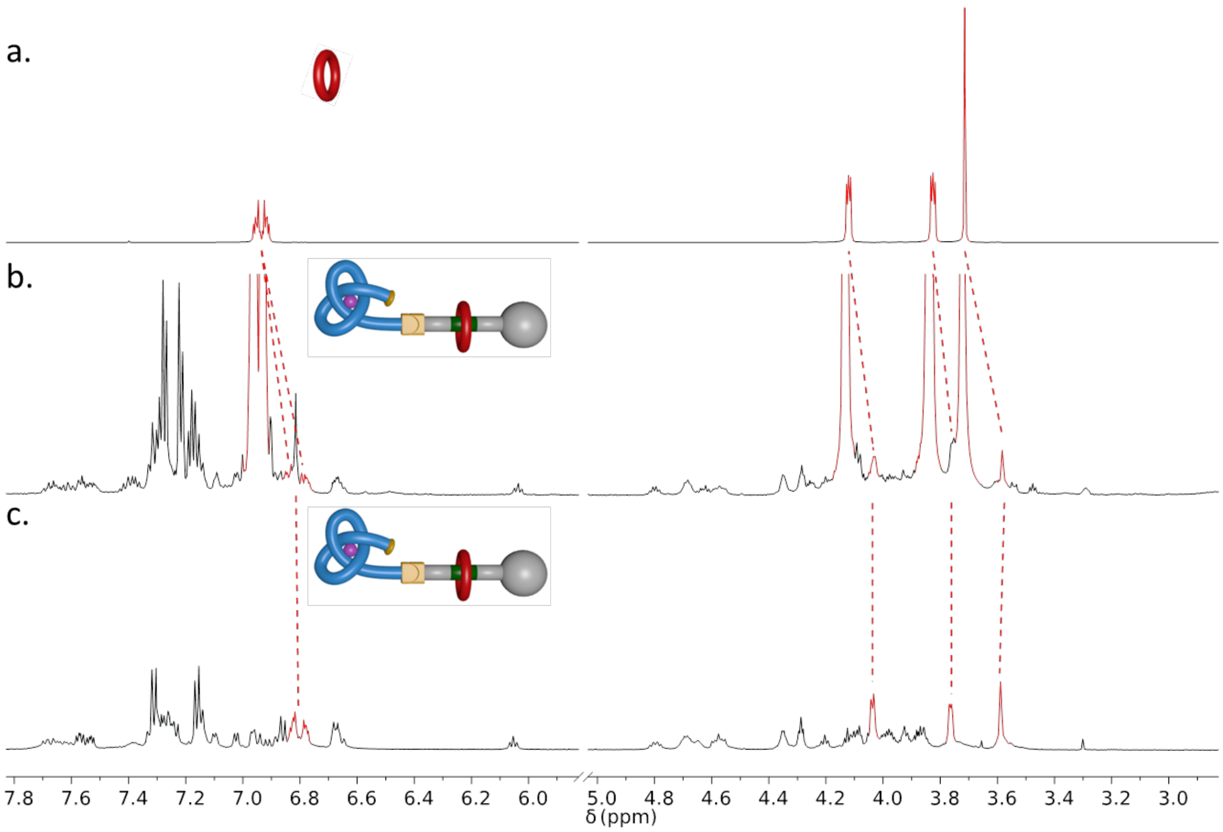

Figure S8. Remetallation of **L1**. (a) Partial ^{1}H NMR (600 MHz, MeCN-*d3*) of DB24C8. (b) Partial ^{1}H NMR (600 MHz, MeCN-*d3*) of crude remetallation reaction mixture. (c) Partial ^{1}H NMR (600 MHz, MeCN-*d3*) of authentic **1**H^{+}.

S3.9 Attempted rethreading of metallated Λ-**L1**H^{+}•Lu

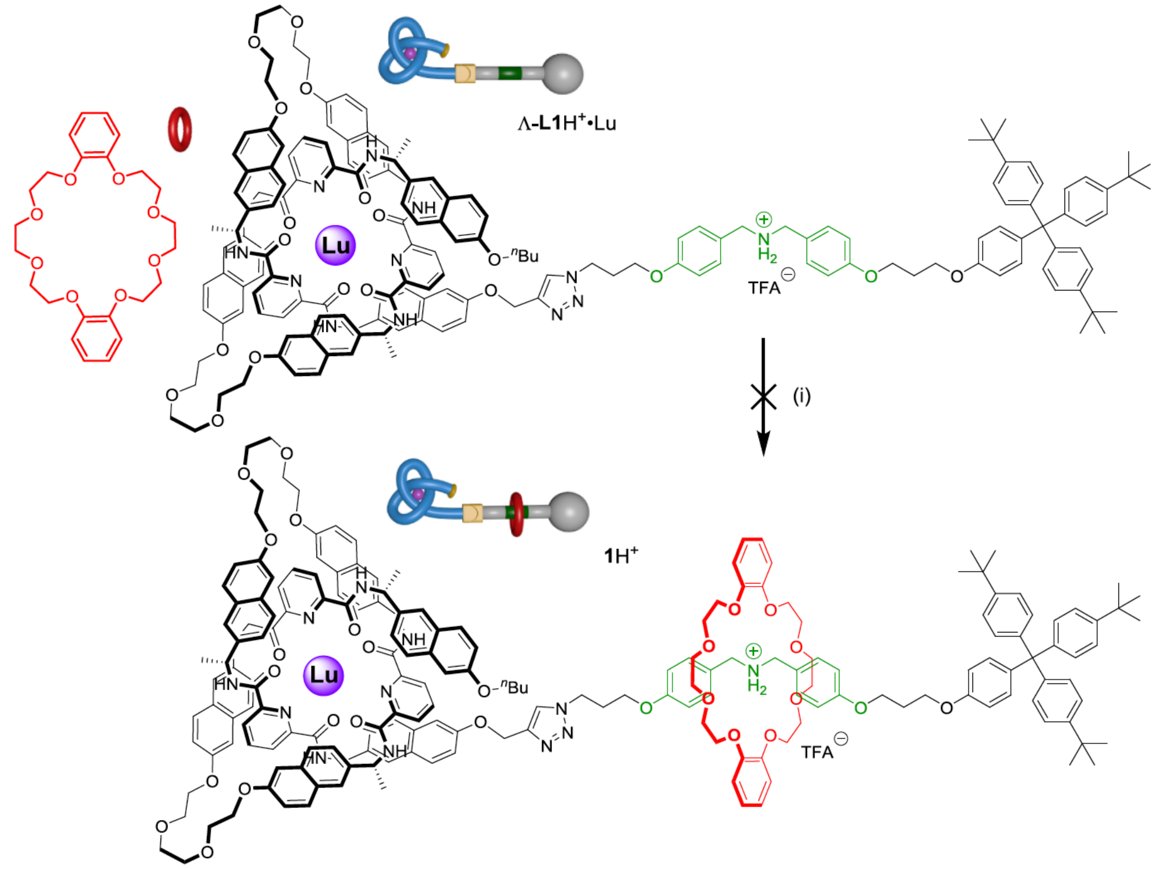

Scheme S9: Attempted threading of Λ-**L1**H^{+}•Lu. Reagents and conditions: (i) DB24C8, MeCN-d_3, r.t., 16 h.

To a solution of Λ-**L1**H^{+}•Lu (1.4 mg) in MeCN-d_3 (0.5 mL) in an NMR tube was added DB24C8 (1.8 mg, 10 eq.) and the threading of the macrocycle was continuously monitored by ^{1}H NMR spectroscopy. No macrocycle was observed to have threaded onto Λ-**L1**H^{+}•Lu after 16 hours.

S4. NMR Spectra

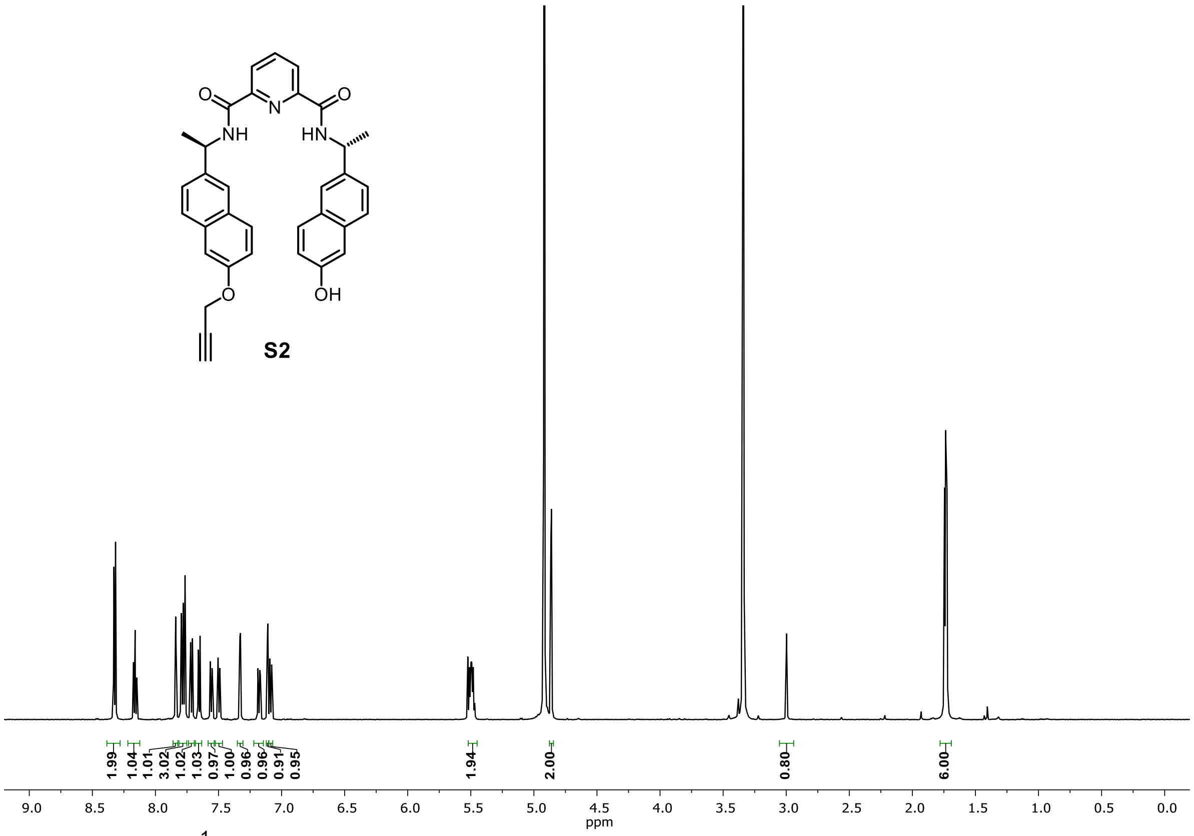

Spectrum S1. ^{1}H NMR (600 MHz, CD_3OD) of **S2**

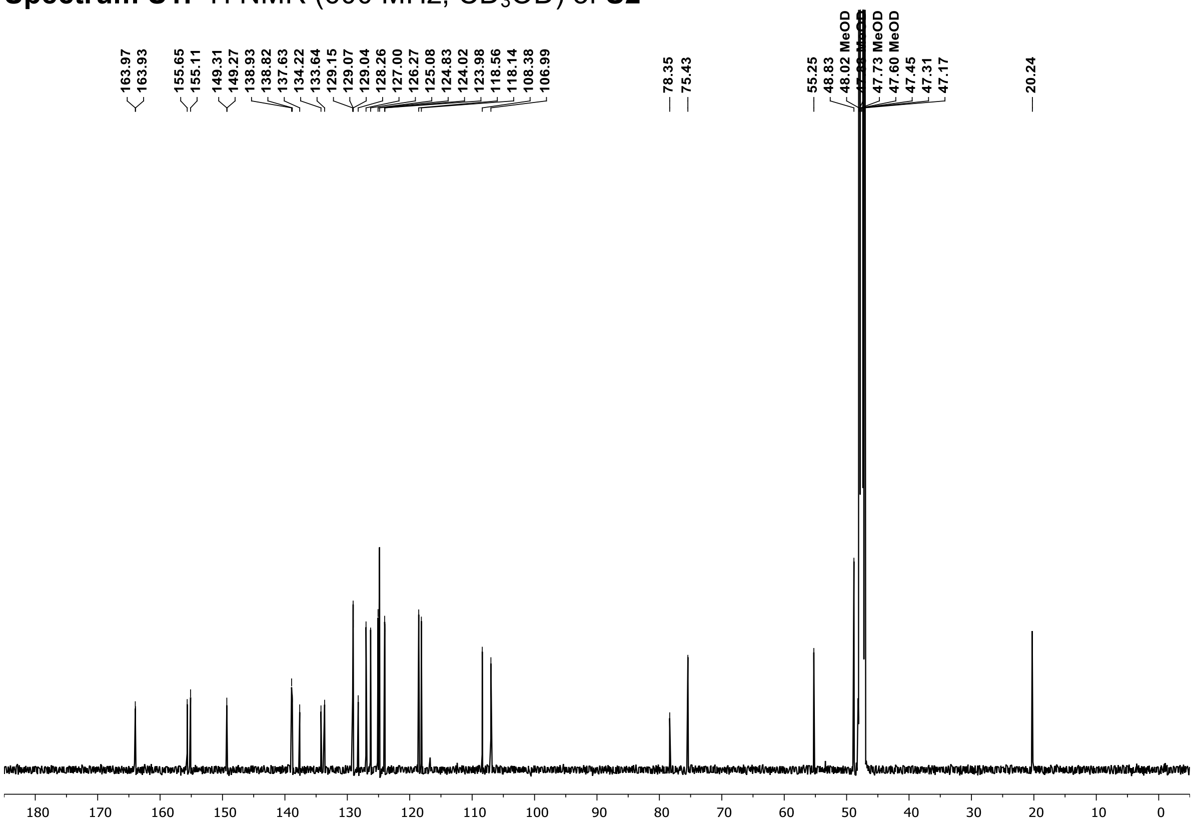

Spectrum S2. ^{13}C NMR (151 MHz, CD_3OD) of **S2**

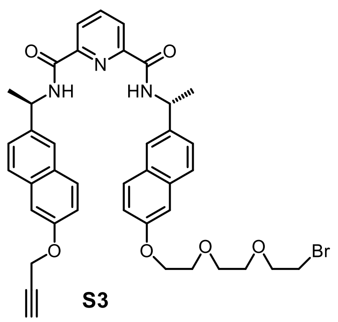

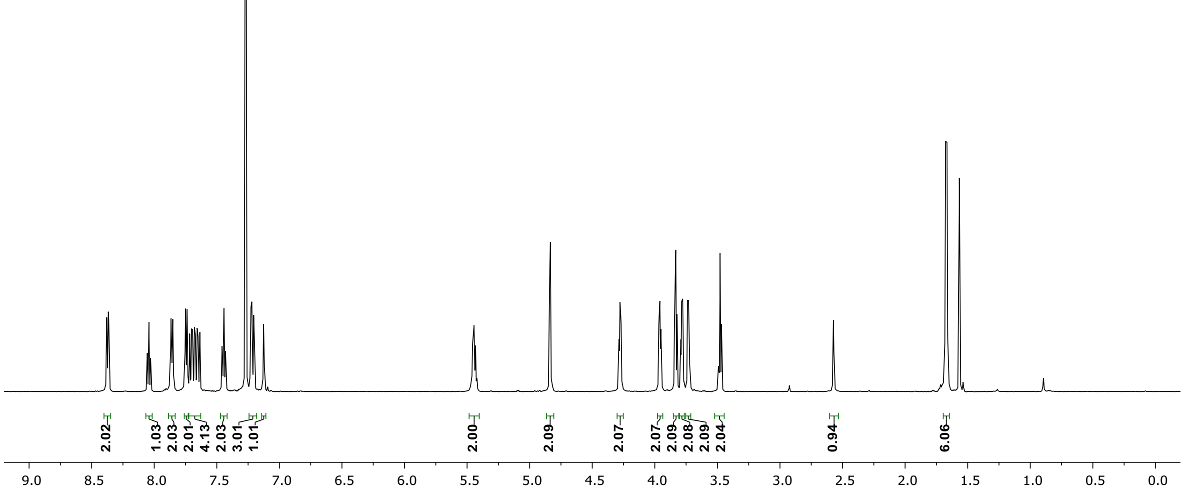

Spectrum S3. ^{1}H NMR (600 MHz, $CDCl_3$) of **S3**

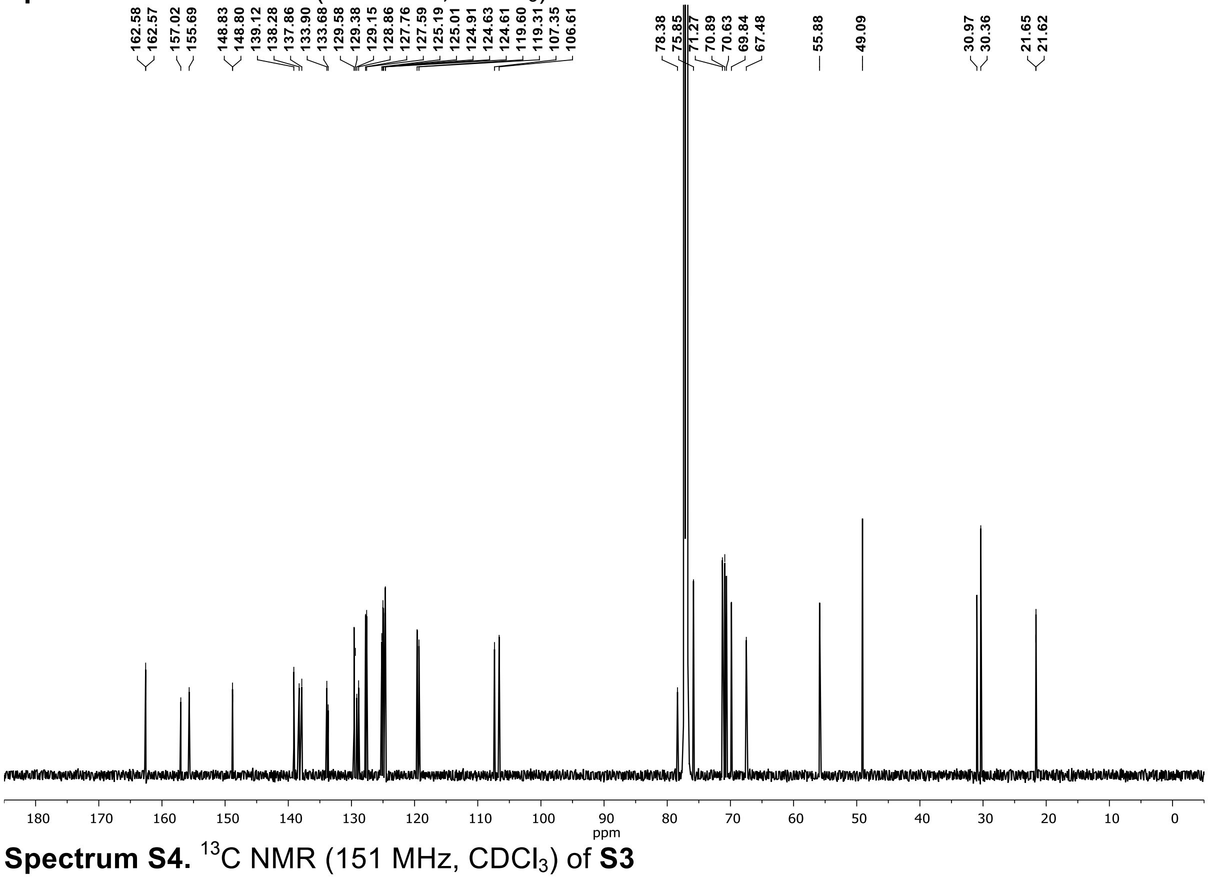

Spectrum S4. ^{13}C NMR (151 MHz, $CDCl_3$) of **S3**

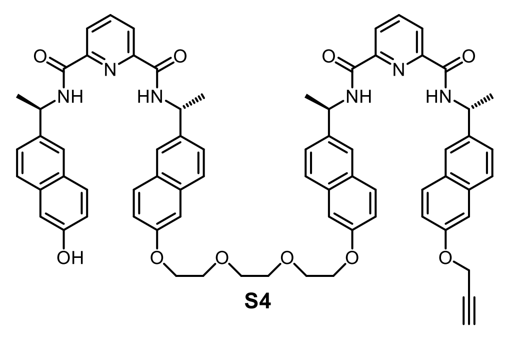

S4

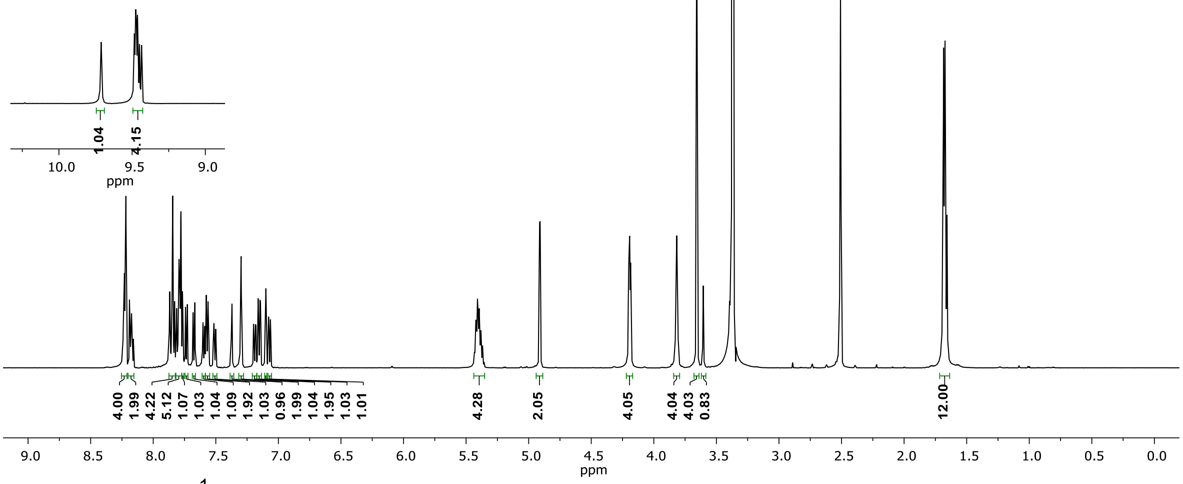

Spectrum S5. ^{1}H NMR (600 MHz, DMSO-d_6) of **S4**

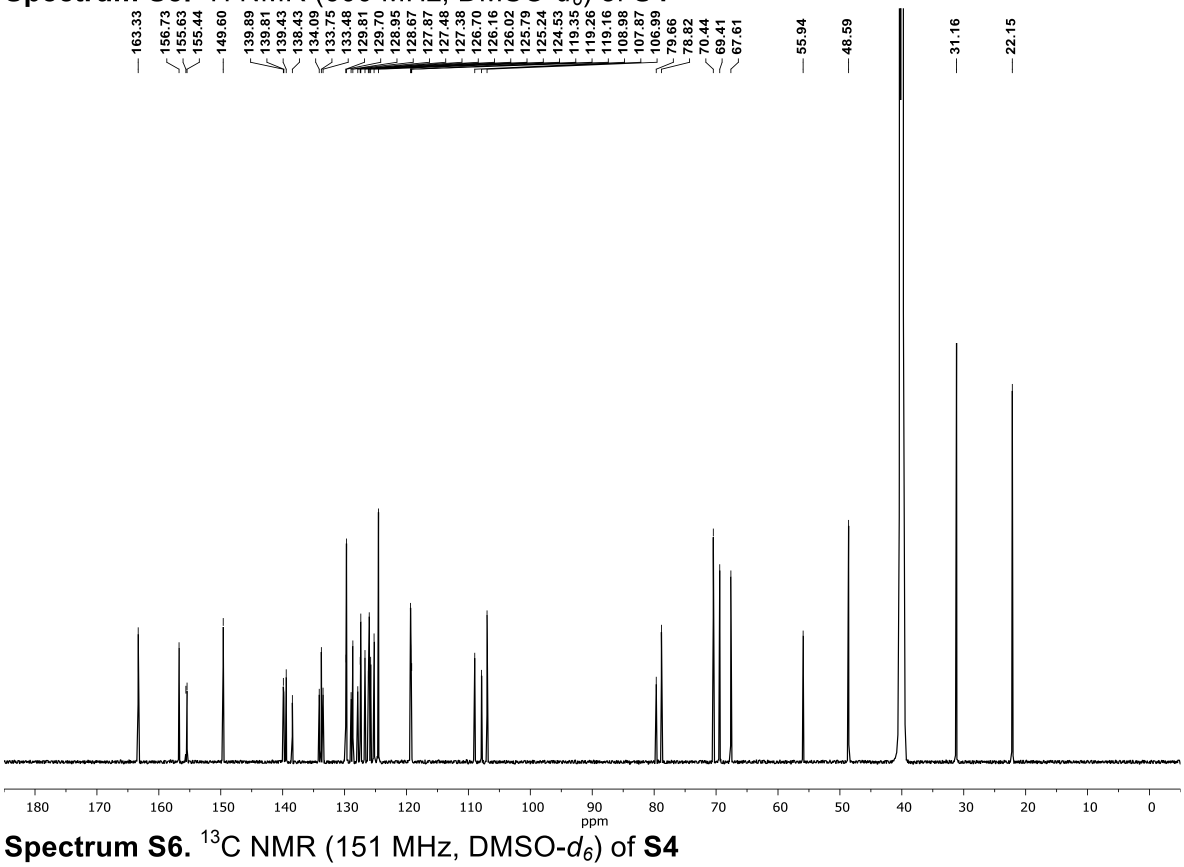

Spectrum S6. ^{13}C NMR (151 MHz, DMSO-d_6) of **S4**

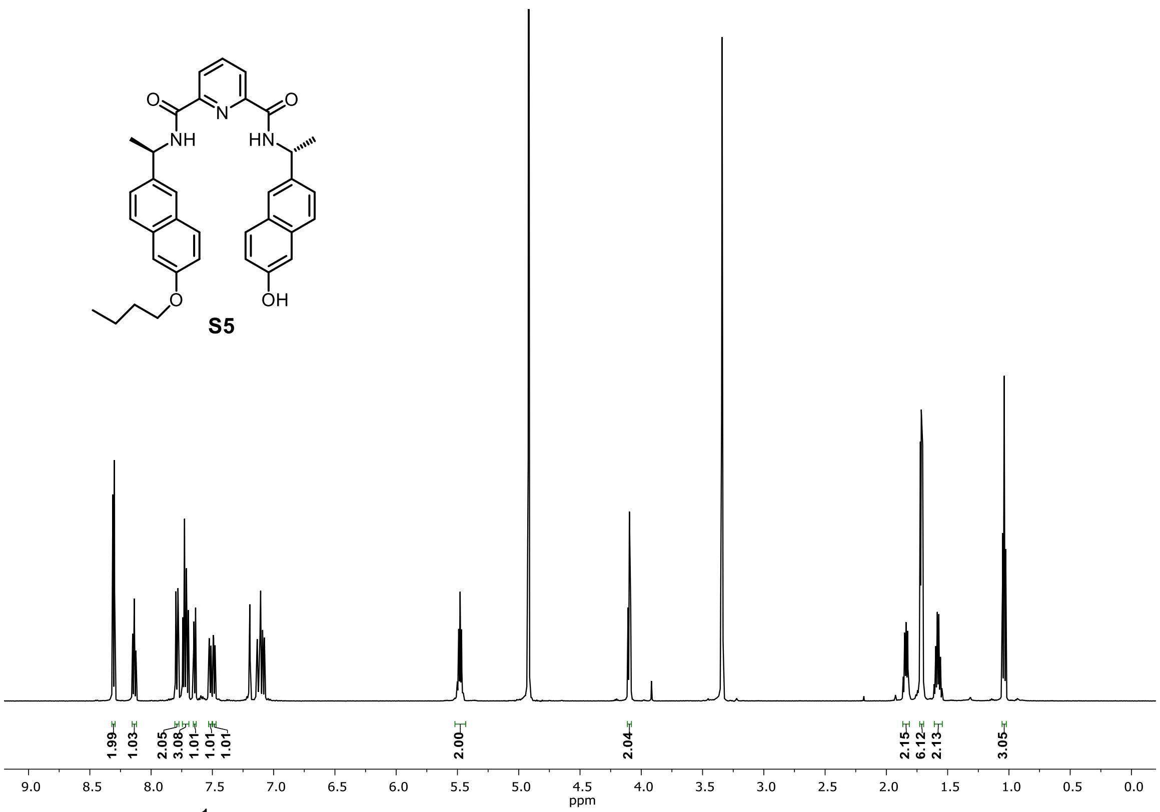

S5

Spectrum S7. ^{1}H NMR (600 MHz, CD_3OD) of **S5**

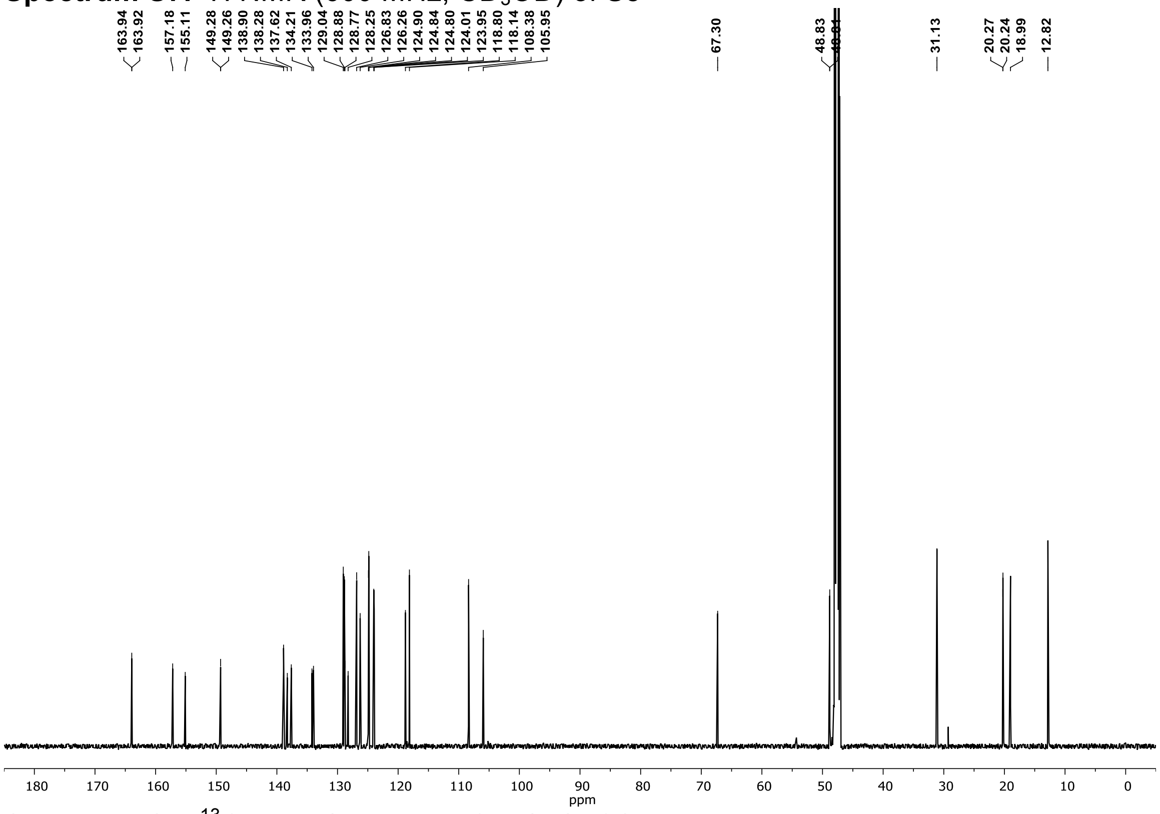

Spectrum S8. ^{13}C NMR (151 MHz, CD_3OD) of **S5**

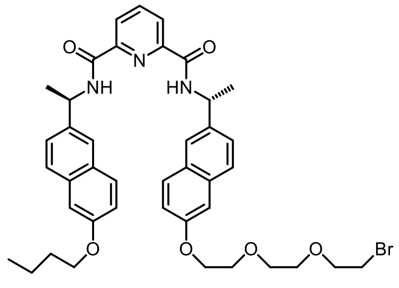

S6

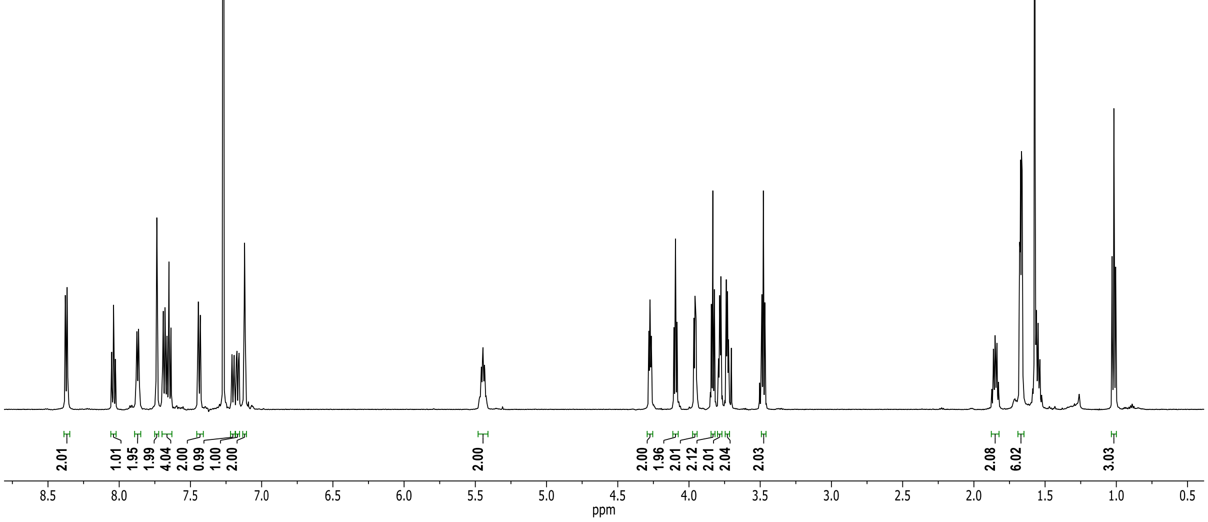

Spectrum S9. ^{1}H NMR (600 MHz, $CDCl_3$) of **S6**

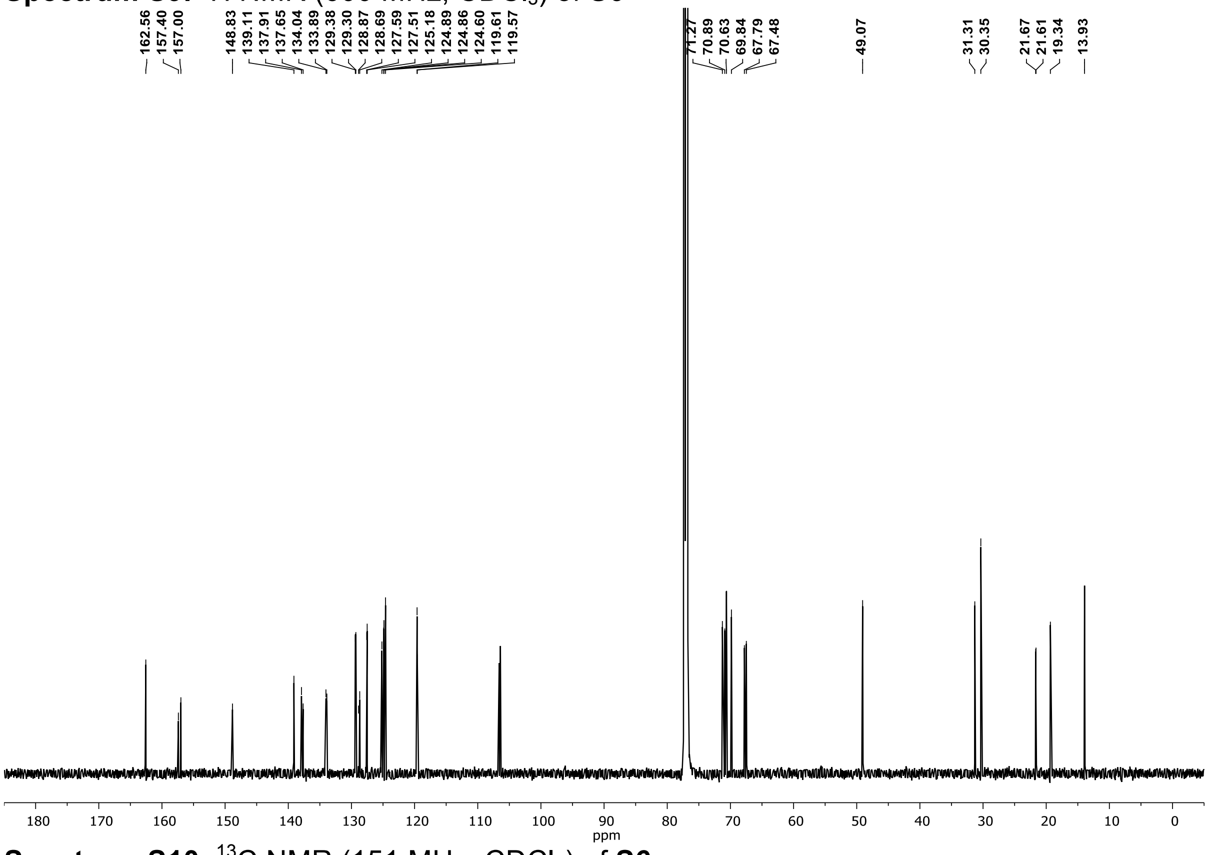

Spectrum S10. ^{13}C NMR (151 MHz, $CDCl_3$) of **S6**

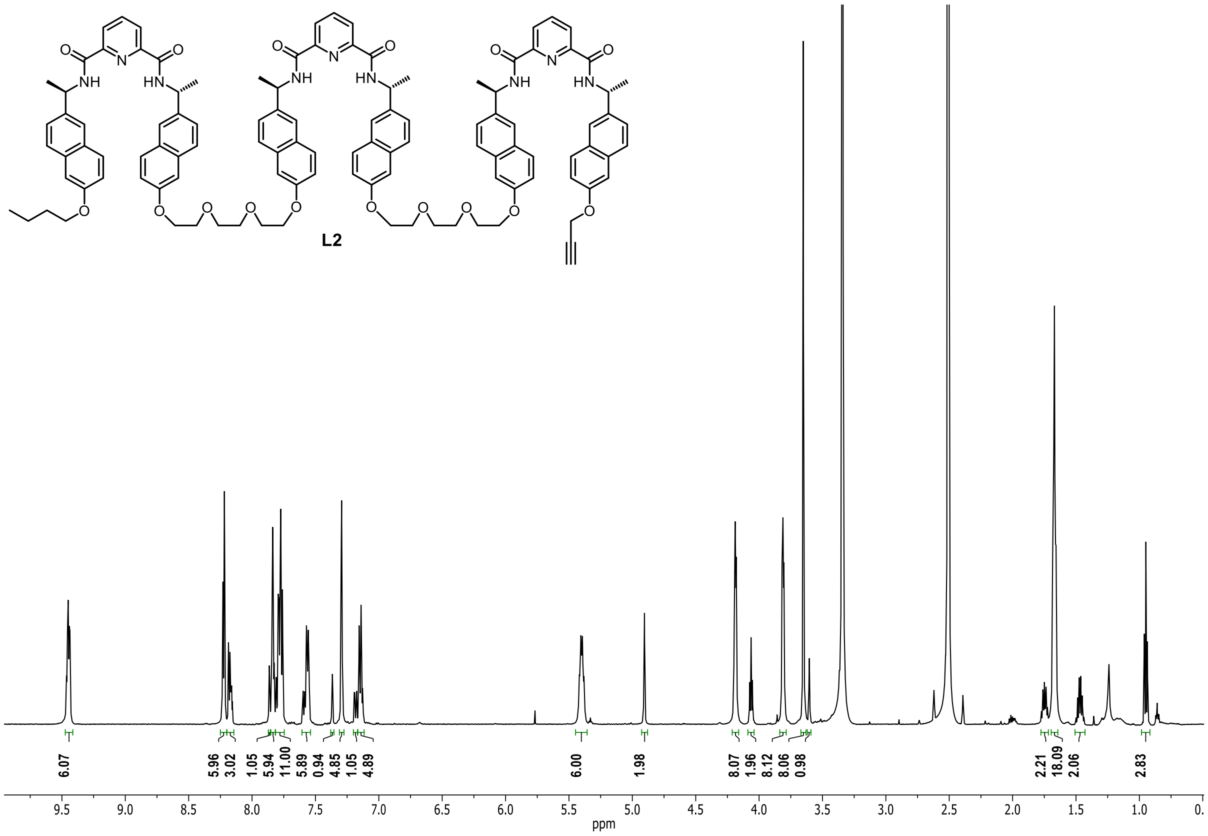

Spectrum S11. ^{1}H NMR (600 MHz, DMSO-d_6) of **L2**

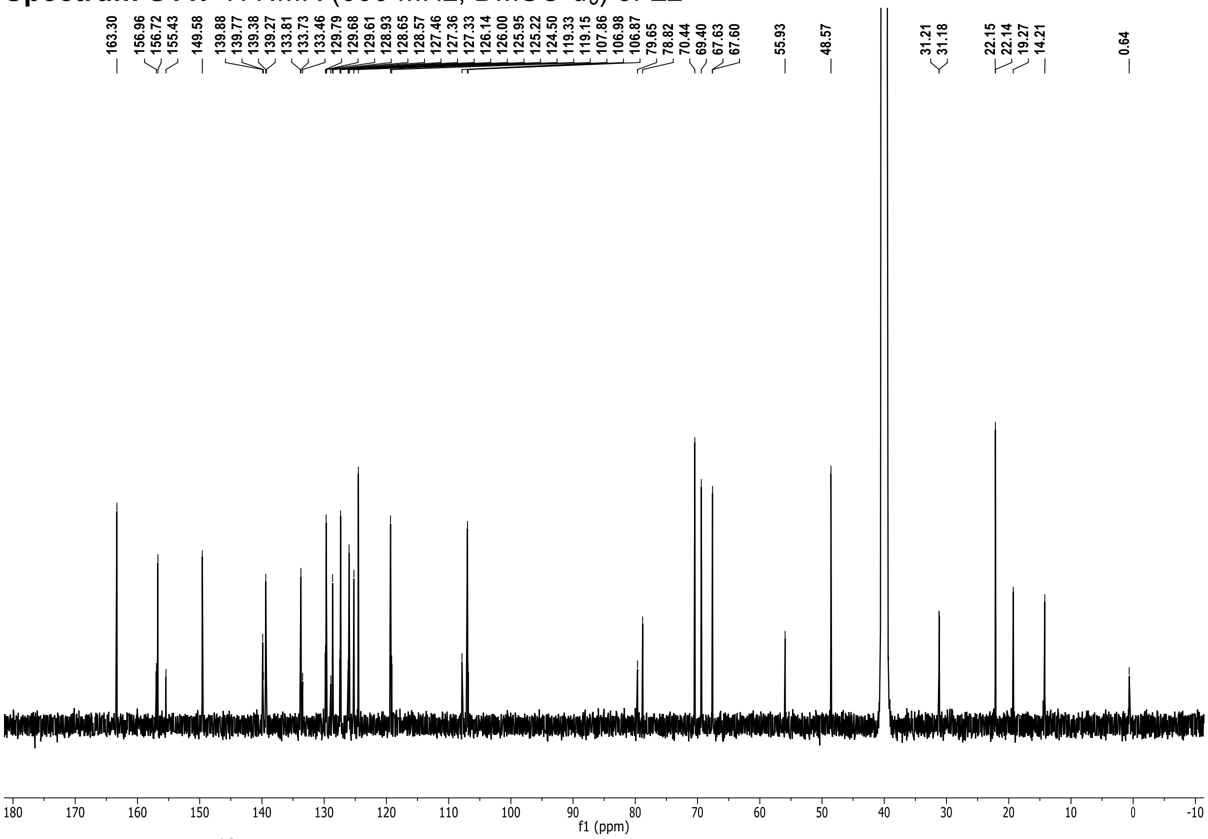

Spectrum S12. ^{13}C NMR (151 MHz, DMSO-d_6) of **L2**

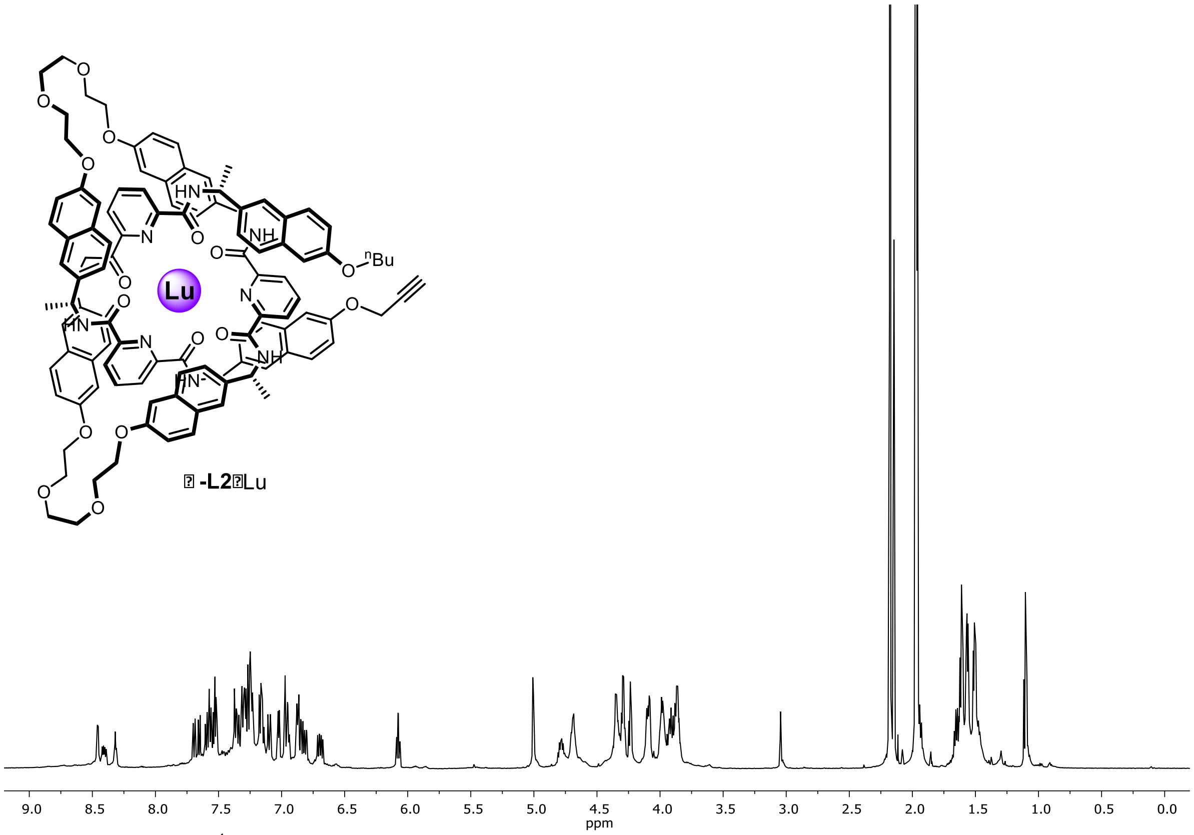

Spectrum S13. ^{1}H NMR (600 MHz, MeCN-d_3) of Λ-**L2**•Lu

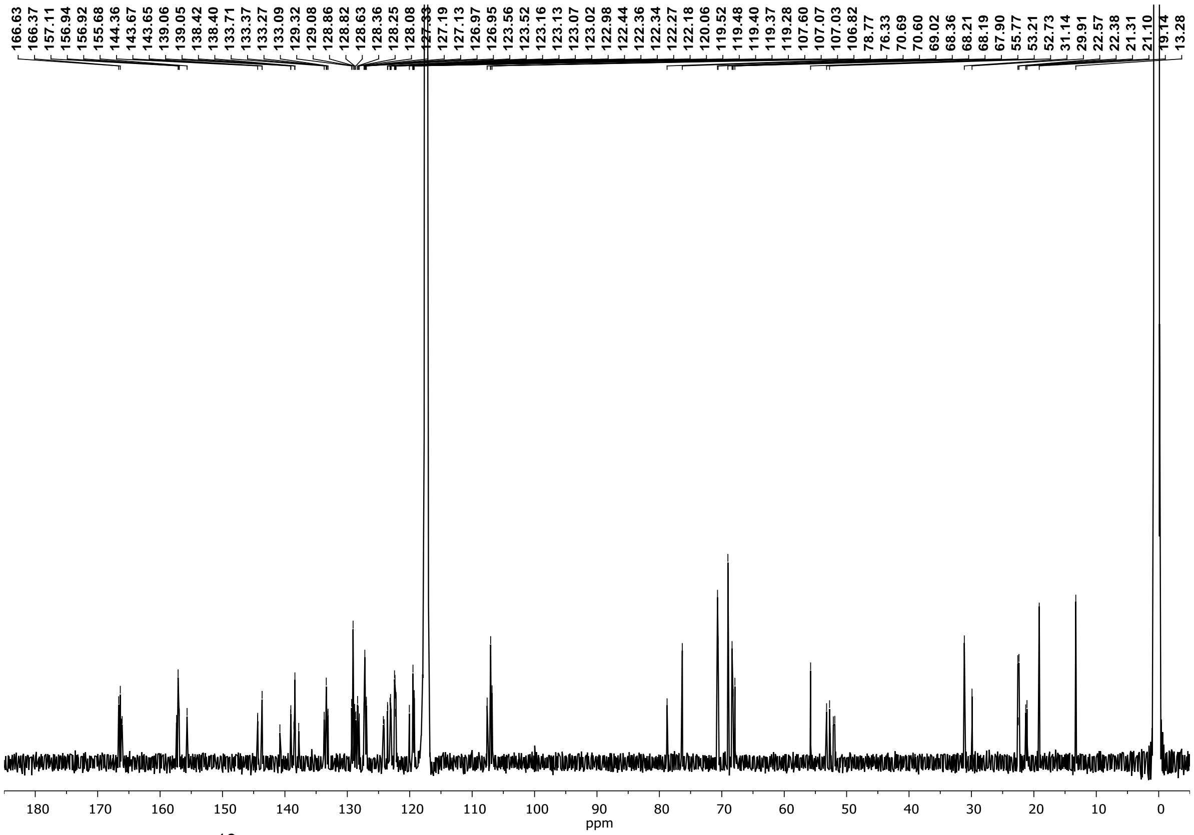

Spectrum S14. ^{13}C NMR (151 MHz, MeCN-d_3) of Λ-**L2**•Lu

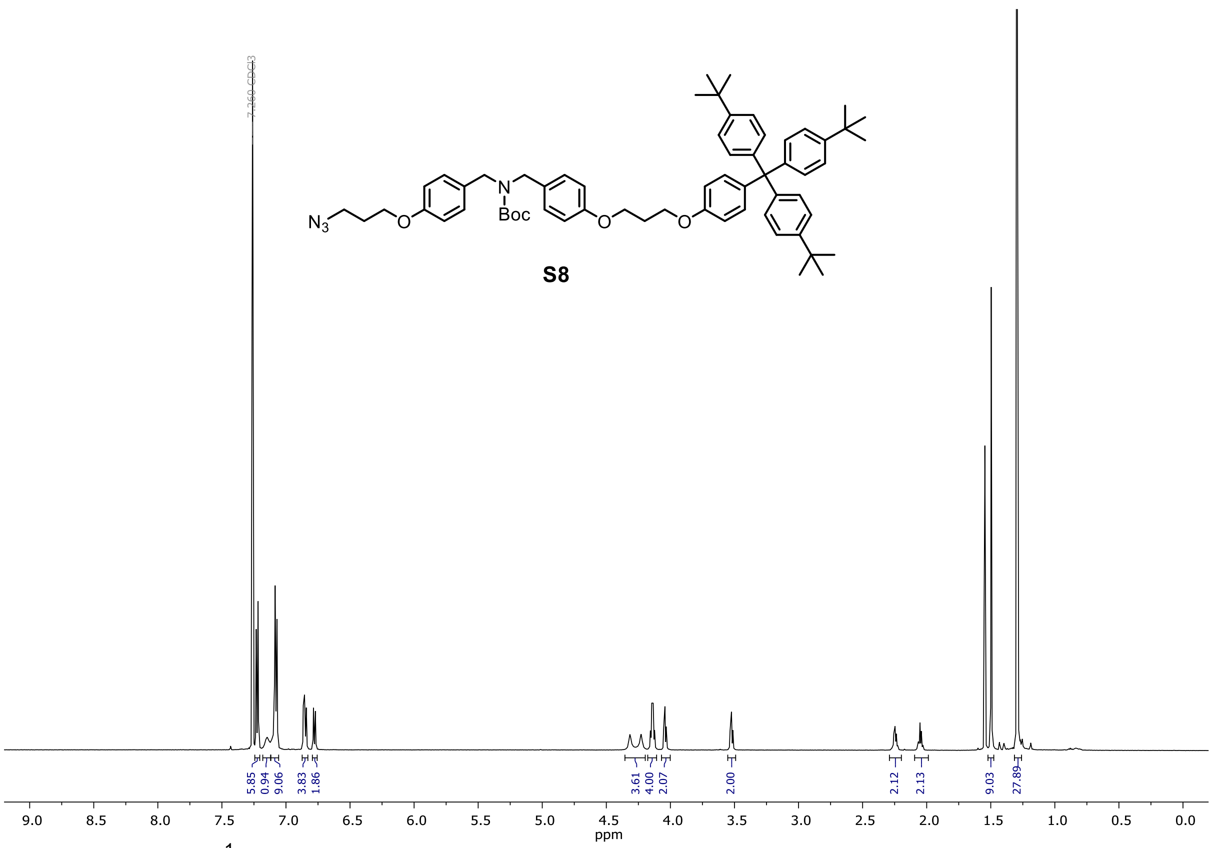

Spectrum S15. ^{1}H NMR (600 MHz, $CDCl_3$) of **S8**

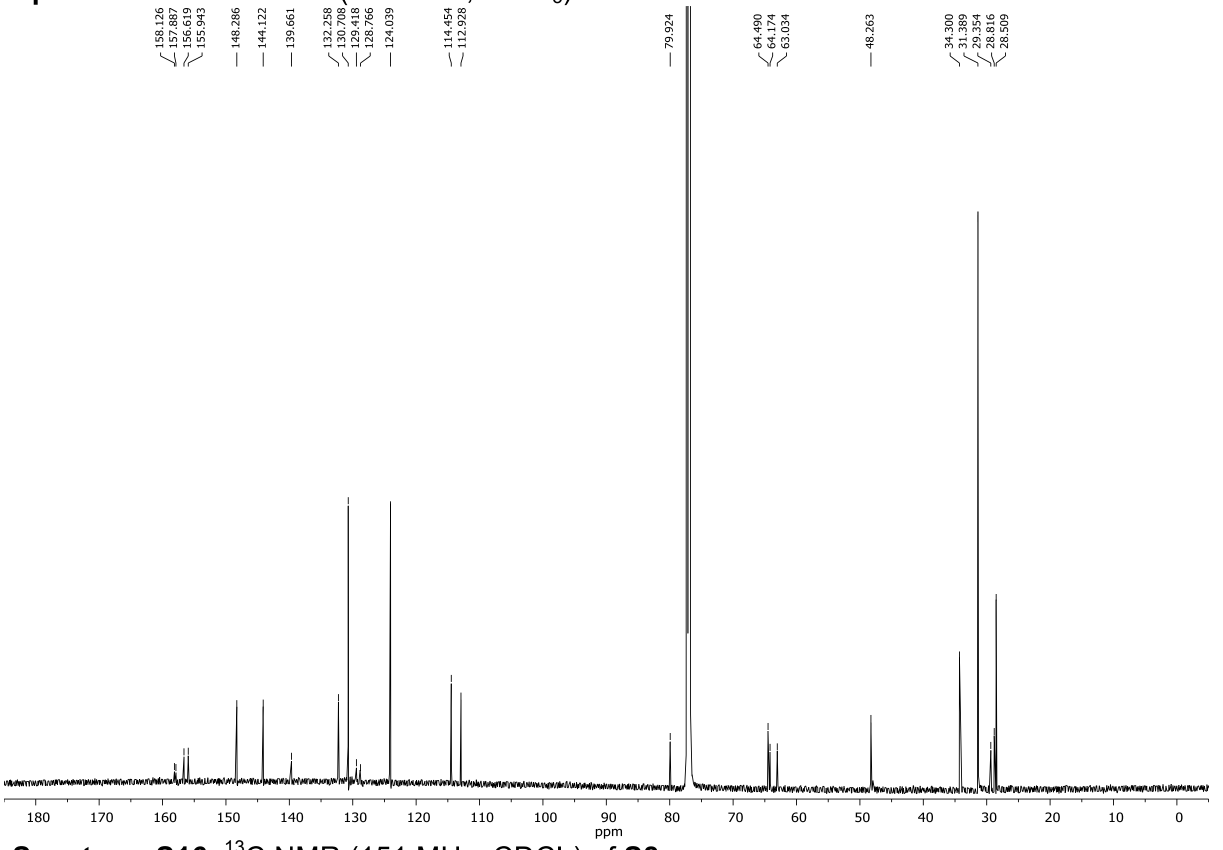

Spectrum S16. ^{13}C NMR (151 MHz, $CDCl_3$) of **S8**

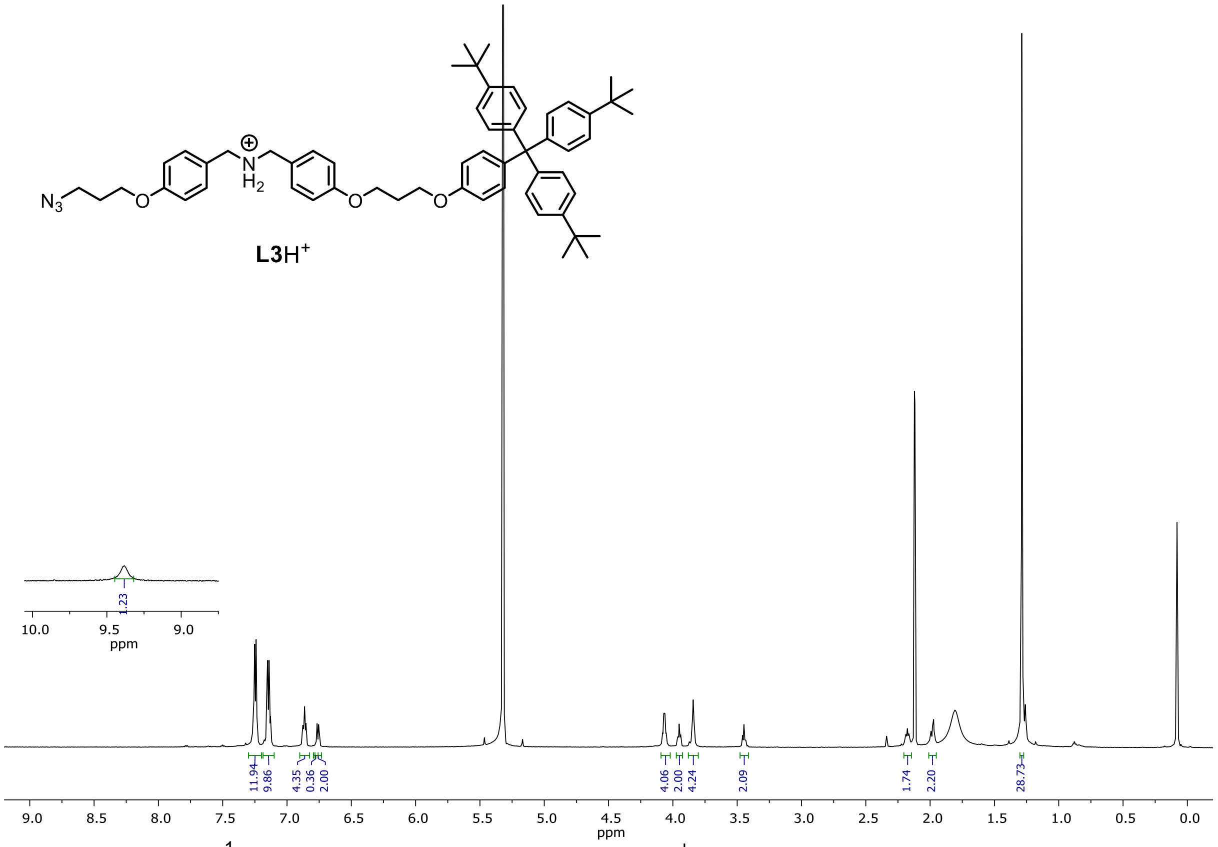

Spectrum S17. ^{1}H NMR (600 MHz, CD_2Cl_2) of **L3**H$^+$

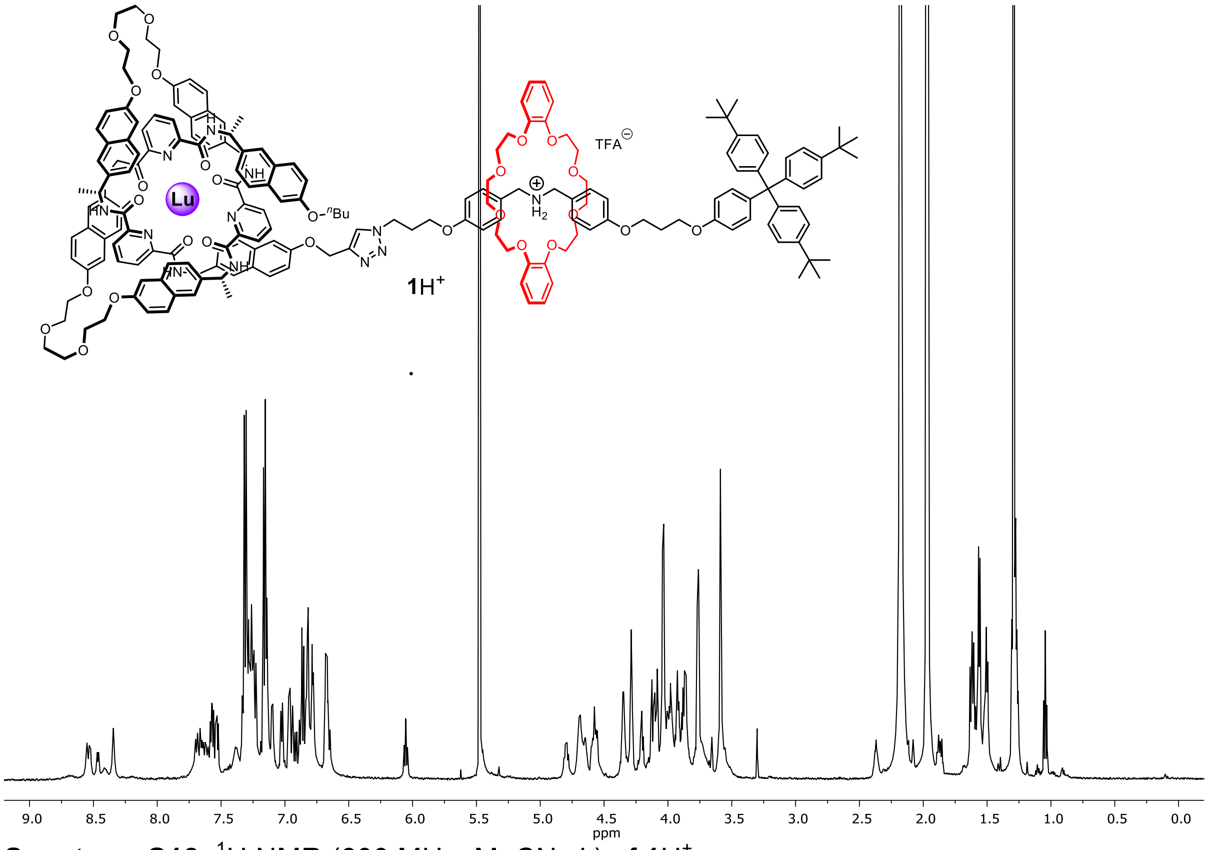

Spectrum S18. ^{1}H NMR (600 MHz, MeCN-d_3) of **1**H$^+$

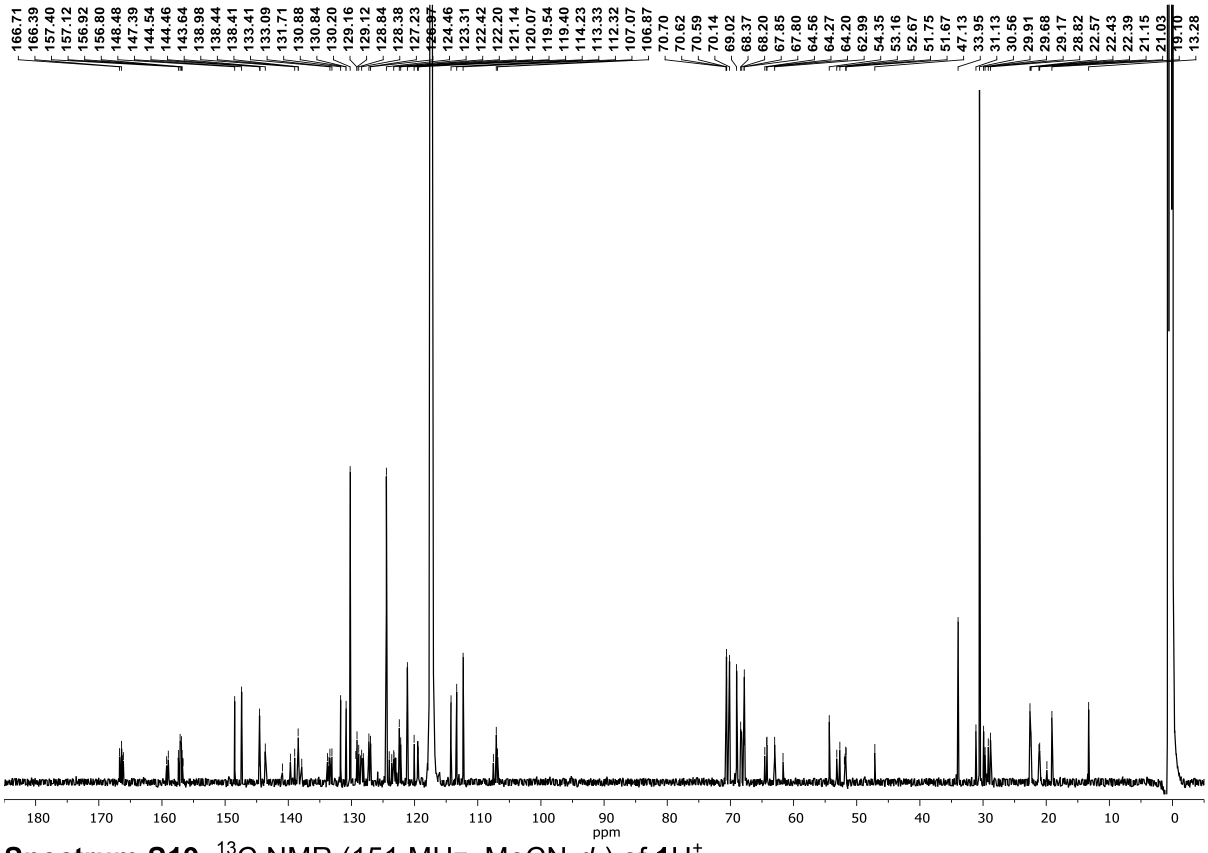

Spectrum S19. ^{13}C NMR (151 MHz, MeCN-d_3) of **1**H$^+$

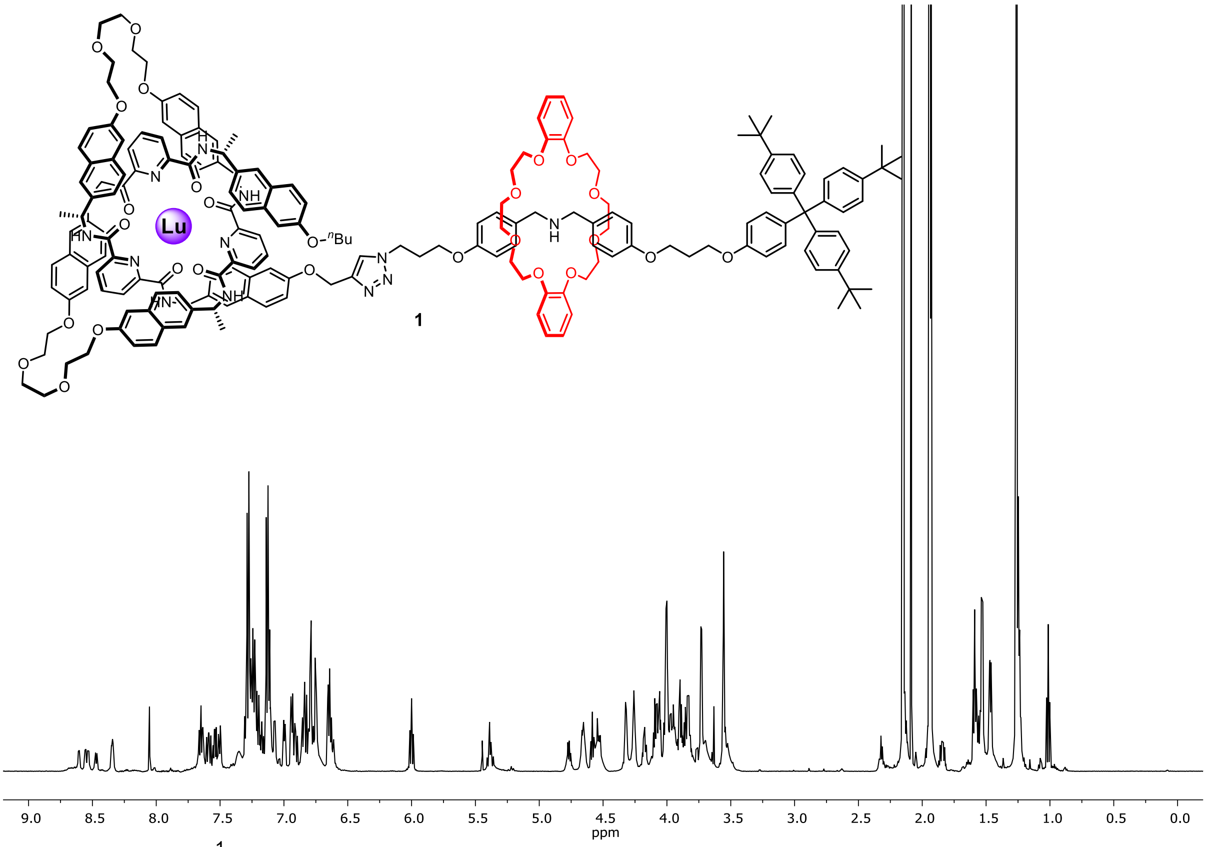

Spectrum S20. ^{1}H NMR (600 MHz, MeCN-d_3) of **1**

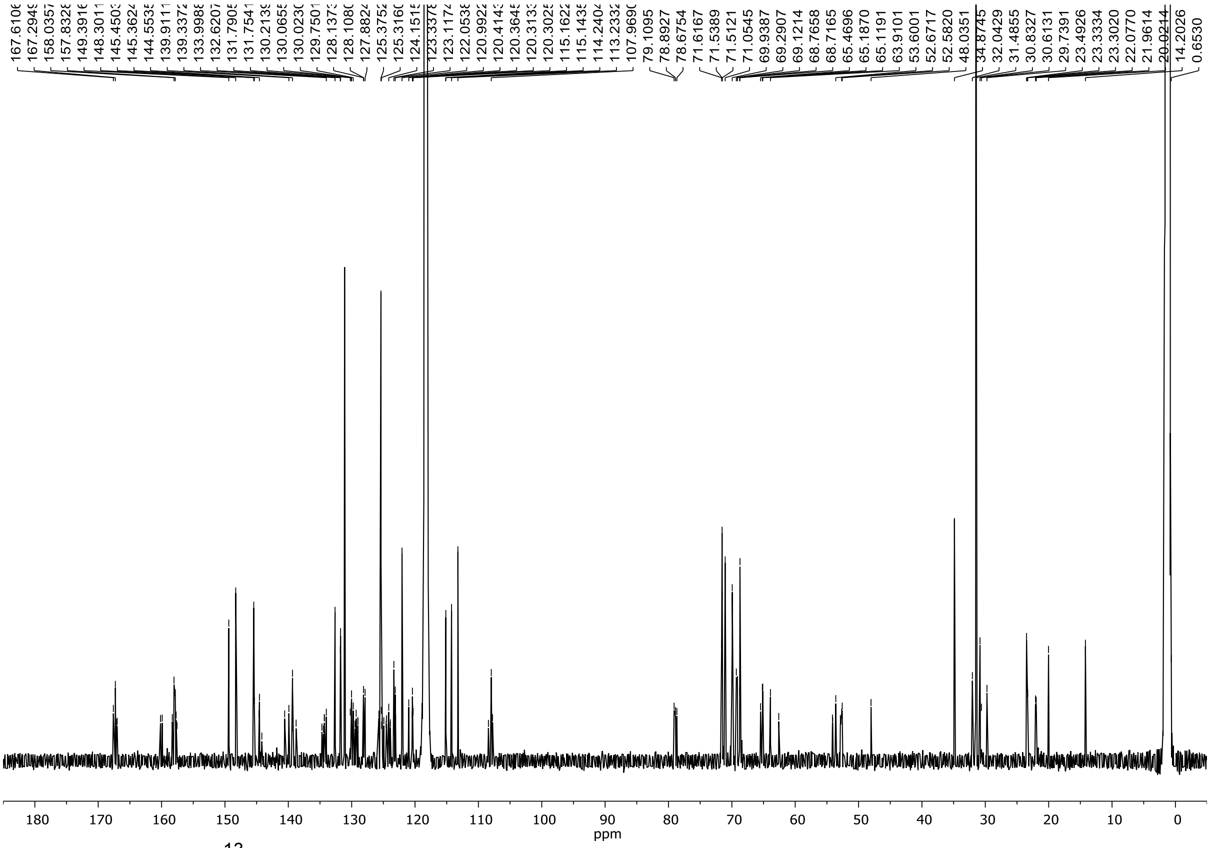

Spectrum S21. ^{13}C NMR (151 MHz, MeCN-d_3) of **1**

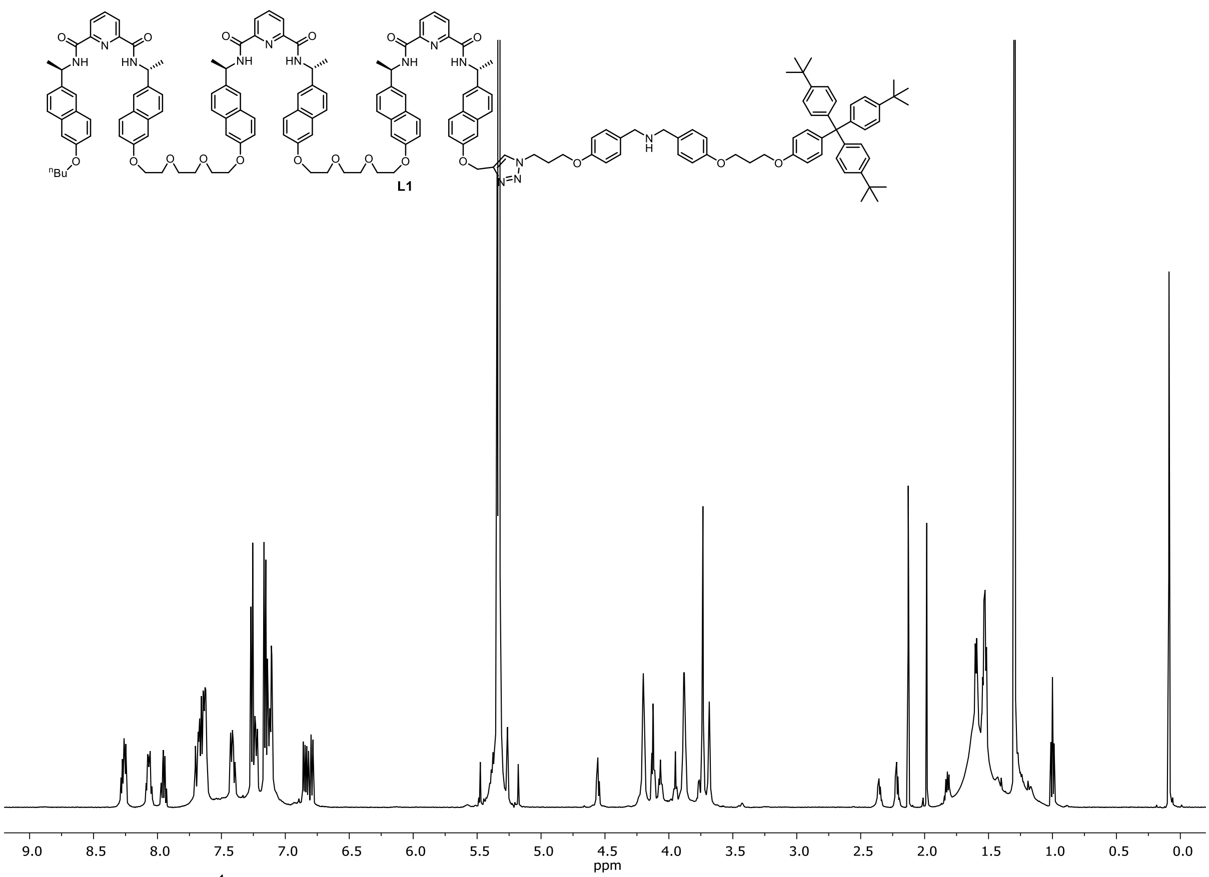

Spectrum S22. ^{1}H NMR (600 MHz, CD_2Cl_2) of **L1**

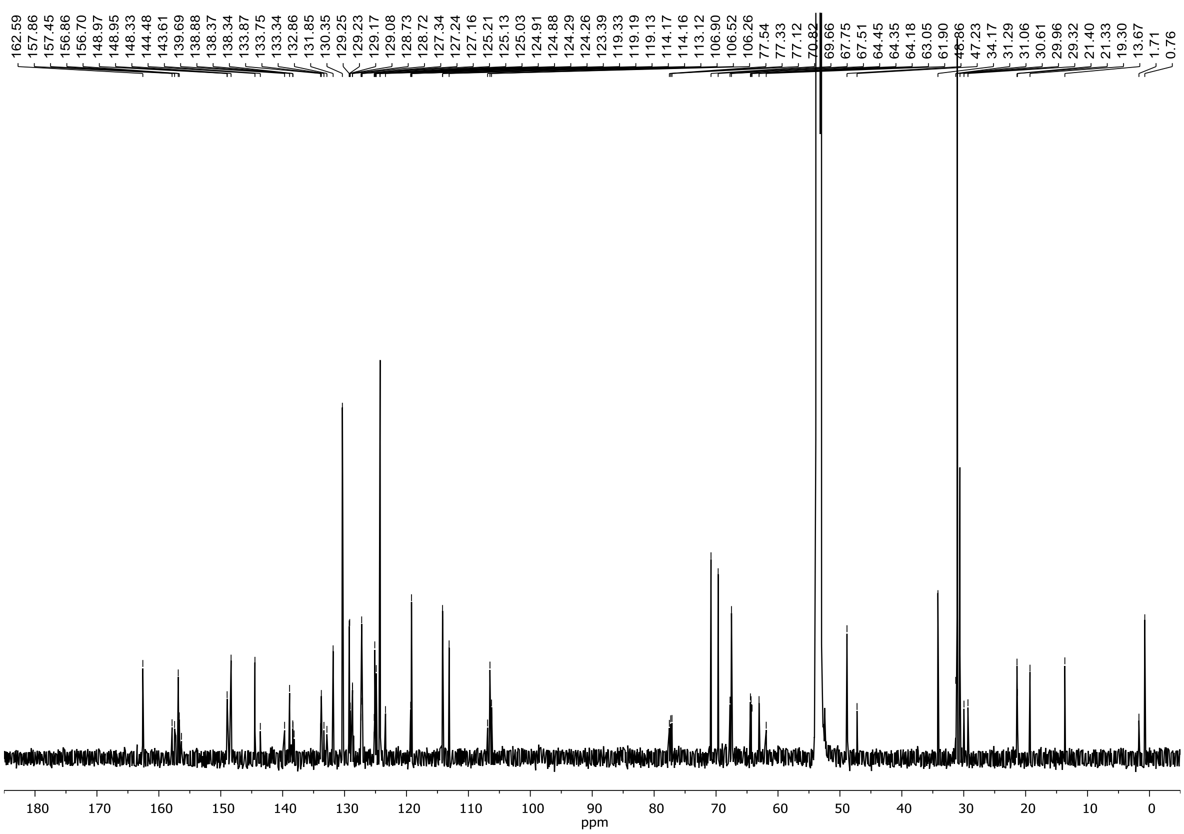

Spectrum S23. ^{13}C NMR (151 MHz, CD_2Cl_2) of **L1**

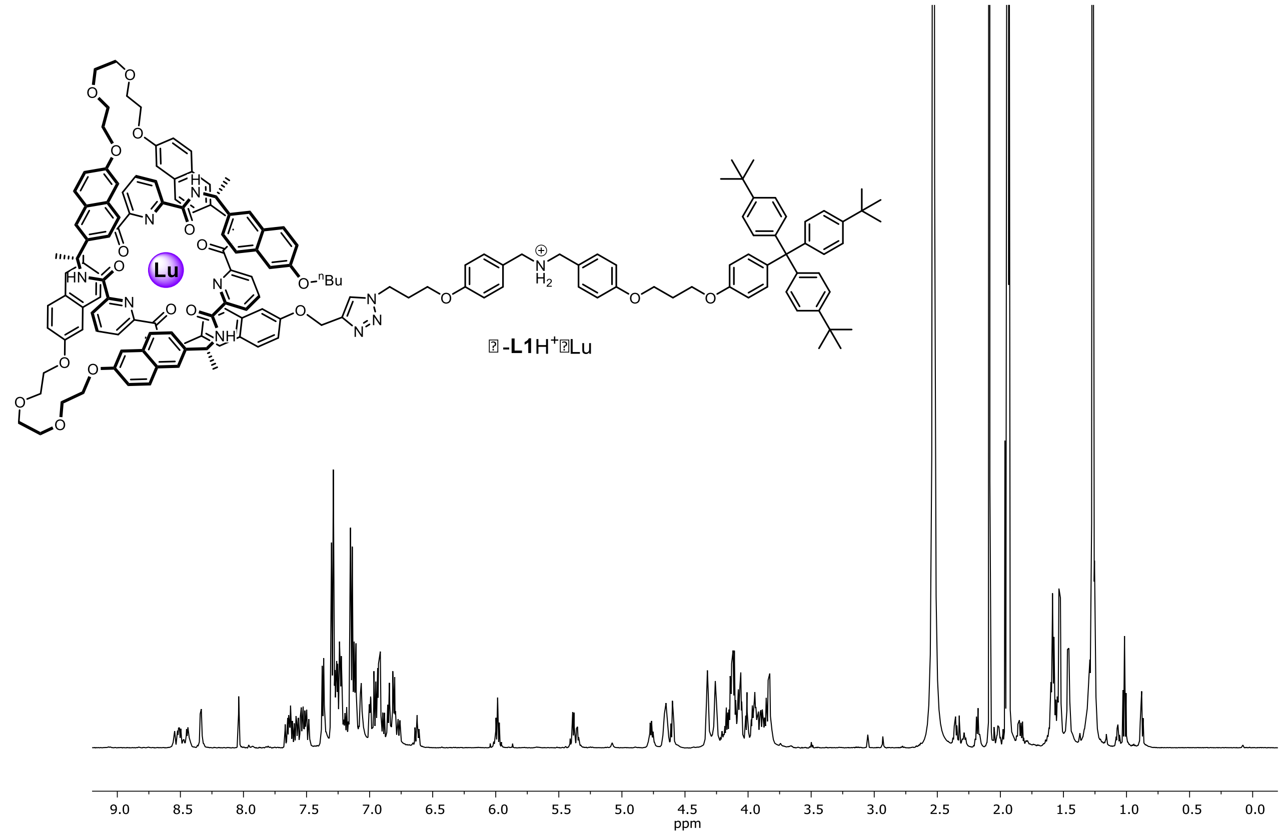

Spectrum S24. ^{1}H NMR (600 MHz, MeCN-d_3) of Λ-**L1**H^{+}•Lu

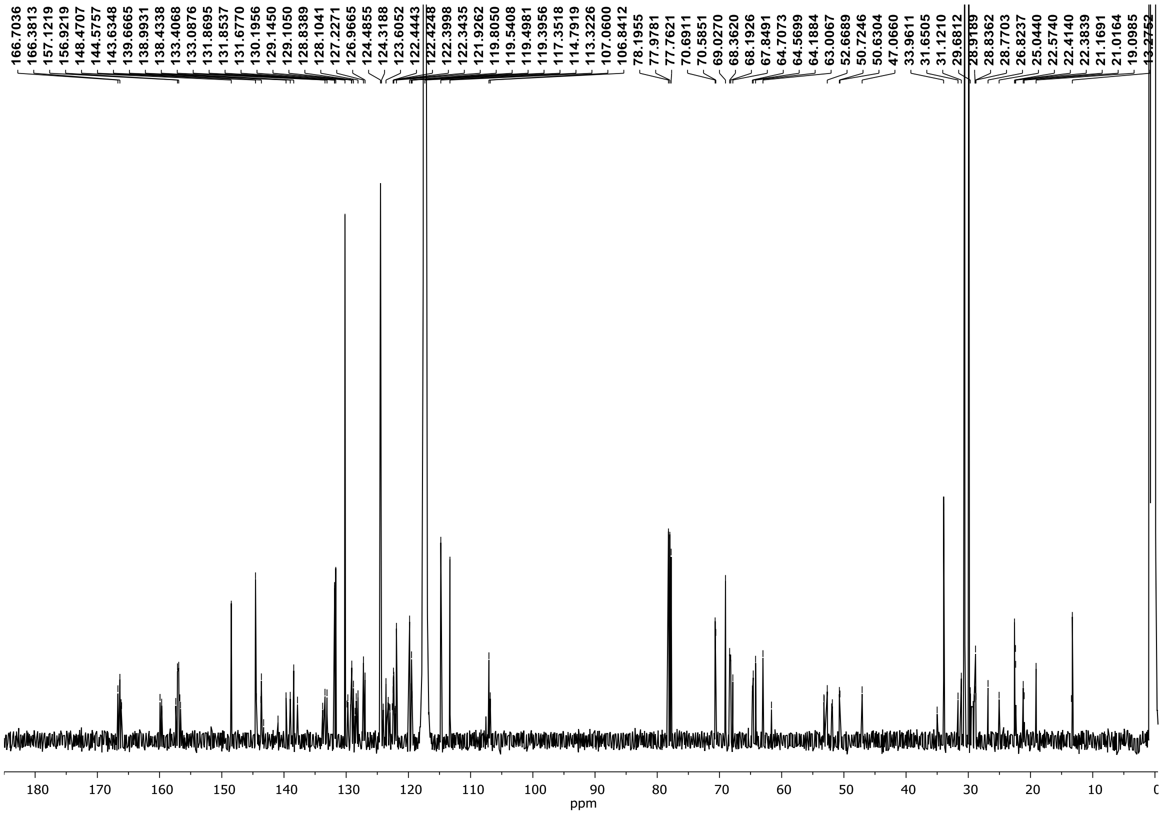

Spectrum S25. ^{13}C NMR (151 MHz, MeCN-d_3) of Λ-**L1**H^{+}•Lu

S5. Supplementary Information References

[1] S. Erbas-Cakmak, S. D. P. Fielden, U. Karaca, D. A. Leigh, C. T. McTernan, D. J. Tetlow, M. R. Wilson, *Science* **2017**, *358*, 340–343.

[2] G. Zhang, G. Gil-Ramírez, A. Markevicius, C. Browne, I. J. Victorica-Yrezabal, D. A. Leigh, *J. Am. Chem. Soc.* **2015**, *137*, 10437–10442.
